# A scoping review of telehealth diagnosis of autism spectrum disorder

**DOI:** 10.1371/journal.pone.0263062

**Published:** 2022-02-10

**Authors:** Katherine Kuhl-Meltzoff Stavropoulos, Yasamin Bolourian, Jan Blacher

**Affiliations:** 1 School of Education, University of California, Riverside, CA, United States of America; 2 Department of Psychology, University of California, Los Angeles, CA, United States of America; University of Wyoming College of Health Sciences, UNITED STATES

## Abstract

**Background:**

Considering the COVID-19 pandemic, understanding the reliability, validity, social validity, and feasibility of using telehealth to diagnose ASD is a critical public health issue. This paper examines evidence supporting the use of telehealth methods to diagnose ASD and outlines the necessary modifications and adaptations to support telehealth diagnosis.

**Methods and procedures:**

Studies were identified by searching PubMed and PsychInfo electronic databases and references lists of relevant articles. Only peer reviewed articles published in English with a focus on using telehealth for the purposes of diagnosing ASD were included. Searches were conducted through June 3^rd^, 2021.

**Outcomes and results:**

A total of 10 studies were identified as meeting inclusion criteria. Of the eight papers that reported on reliability (e.g., accuracy), telehealth methods to diagnose ASD were between 80–91% accurate when compared with traditional in-person diagnosis. Six studies reported on validity (i.e., sensitivity and/or specificity). All six studies calculated sensitivity, with values ranging from 75% and 100%. Five of the six studies calculated specificity, with values ranging from 68.75% and 100%. The seven papers that reported social validity indicated that caregivers, as well as adult participants and clinicians, were mostly satisfied with telehealth. Feasibility was reported by seven studies and suggests that telehealth methods appear largely viable, though some challenges were reported.

**Conclusions and implications:**

Although findings reviewed here are promising, more research is needed to verify the accuracy, validity, and feasibility of utilizing telehealth to diagnose ASD. Studies with larger sample sizes and samples across sites will be critical, as these will allow clinicians to identify subjects most likely to benefit from telehealth as well as those more likely to require an in-person assessment. This research is important not only due to the current pandemic, but also due to increased prevalence rates of ASD and an insufficient number of diagnostic providers—particularly in rural and/or otherwise under-served communities.

## Introduction

Telehealth has been promoted as a viable means to deliver healthcare, information, and intervention to individuals with autism spectrum disorder (ASD) and their families. While telehealth has been shown to be instrumental in reaching rural and remote areas, the use of telehealth to support long-distance diagnostic evaluations of ASD has received less consideration and research. While ASD is distinctly defined in the Diagnostic and Statistical Manual (DSM) of the American Psychiatric Association (American Psychological Association [APA], 2013), autistic children are heterogeneous in their presentation of symptoms, characteristics, behaviors, and intelligence. It requires well-trained professionals to screen and diagnose. Essentially, ASD is a neurodevelopmental disorder characterized by impairments in social-communication and the presence of restricted interests and/or repetitive behaviors [1]. Thirty years ago, autism was considered rare, with 1 in 2,500 children diagnosed. Today, the Center for Disease Control and Prevention estimates that 1 in 54 children are diagnosed [2]. While there is no known cure for ASD, intensive, early intervention has proven efficacious [3]. Furthermore, there is a window of opportunity in early childhood that can be exploited to assure optimal development. However, not all families can access services and take advantage of this developmental opportunity.

One important barrier in accessing intervention relates to receiving a timely diagnosis. Simply put, persons who experience delays in diagnosis might not receive the intervention support that they need. Within rural areas, the shortage of service providers who conduct early screening and diagnosis puts entire communities at risk of not having children with ASD identified within the developmental window to maximize intervention effects [4, 5]. In addition, ethnic disparities are evident in autism-related services. On average, children from Latinx families are diagnosed later than children from non-Latinx families [2]. Note that we use the term “Latinx” in order to be gender inclusive when referring to persons of Latin American descent, traditionally referred to as Latino or Latina. Thus, there is increasing recognition that early identification and diagnosis are particularly needed for groups who might otherwise not receive it due to their geographic location, socioeconomic status, ethnicity, or a combination of these factors. Telehealth has been discussed as a potential delivery method for diagnostic services which could increase access to communities at risk. Moreover, due to the coronavirus disease 2019 (COVID-19), the need to bolster evidence regarding the use of telehealth diagnostic evaluations has escalated. This scoping review explores reports of accuracy, validity, and feasibility of telehealth methods for diagnosing ASD.

### Telehealth and the rise in remote services

Tele-mental health or telehealth is defined by the American Psychological Association as “the provision of behavioral and/or mental health care services using technological modalities is lieu of, or in addition to, traditional face-to-face methods” [6]. The two most common telehealth methods are videoconferencing and store-and-forward methods. Videoconferencing involves real-time, live interactions and/or observation conducted via a videoconferencing service (e.g., Zoom, Skype, Facetime). Store-and-forward methods involve having a caregiver record various scenarios with their child or adolescent and uploading them to a server where they are stored and forwarded to a clinician.

There is increasing interest in the utility of using telehealth methods to conduct assessments for diagnosis in a variety of psychiatric conditions. A meta-analysis of fourteen studies (*N* = 500 patients [7]); found that patient satisfaction based on objective assessments via videoconference and in-person services for standardized clinical interviews (e.g., Structured Clinical Interview for the DSM-IV; SCID [8]); was similar. In addition, policy guidelines released by the American Telemedicine Association state that telemedicine is an effective alternative for in-person assessments and diagnosis [9]. These studies provide evidence for the utility of telehealth as a vehicle for assessment and diagnosis broadly.

In the context of ASD, many studies have focused exclusively on the feasibility and effectiveness of using telehealth to provide a range of evaluation and intervention services, including functional behavior assessments [10], early intervention [11], cognitive-behavioral intervention [12], parent training [13, 14], provider training [15, 16], and family support groups [17]. Less is known about how telehealth can be utilized for the diagnosis of ASD.

In light of the COVID-19 pandemic, the importance of telehealth–from screening and diagnosis to delivery of interventions–has increased substantially. Many in-person clinics temporarily closed due to concerns about COVID-19 transmission. Even as some sectors of the economy begin to re-open, particularly those which can operate outdoors, some clinics may remain closed, operate remotely, or utilize hybrid models due to the increased susceptibility to COVID-19 for certain populations, including individuals with ASD and related developmental disabilities [18]. Furthermore, many telehealth clinics are likely to remain in operation long after the pandemic, due in part to their realized potential. Thus, it is more important than ever to review existing literature related to telehealth in ASD to synthesize best practices, highlight commonly faced issues, and provide guidance. The current review will focus on the accuracy, social validity, and feasibility of using telehealth to diagnose ASD. Based on findings from identified studies, the review will also summarize necessary adaptations of telehealth assessments.

### Previous reviews on telehealth for ASD diagnosis

Previous reviews have synthesized existing literature on telehealth as a delivery method for both assessments and intervention services for the ASD population (e.g., [19–30]. All reviews published between 2010 and 2019 focused on the utility of telehealth for diagnostic and intervention services for individuals with ASD. Below, we highlight those reviews that focused exclusively on the use of telehealth to assess or diagnose ASD.

A review of seven articles on telehealth assessments [22] identified four studies that designated young children as “at risk” of ASD, while three studies used telehealth to directly diagnose ASD [31–33]. Notably, two of the three studies using telehealth to diagnose ASD [32, 33] utilized machine learning algorithms rather than clinicians to categorize children as having ASD or not. The third study [31] utilized store-and-forward methods. Parents were instructed on how to record videos of their children engaging in specific behaviors and then upload the videos to a secure server for clinicians to watch and score.

Alfuraydan and colleagues reviewed how various telehealth approaches have been used to diagnose ASD in both children and adults [26]. Of the ten studies reviewed, six utilized telehealth to diagnose ASD remotely, using store and forward methods or “live” video conferencing [31, 34–38]. The other four presented findings from pilot/feasibility studies of remote diagnostic assessments or retrospectively measured the effect of telehealth on caregivers’ ability to access diagnostic evaluation and/or services [39–42].

More recently, one review was published on the utility of telehealth in diagnosing ASD. Berger and colleagues (2021) published a review concerning the use of telehealth to diagnose ASD in young children, specifically between the ages of 12–36 months [28]. This review focused exclusively on synchronous, or “live”, telehealth methods (e.g., real-time interactions) rather than store-and-forward methods (e.g., parents take videos of targeted scenarios which clinicians subsequently reviewed). The authors reviewed five synchronous telehealth methods for assessing ASD: (1) The *ASD Diagnostic Interview and Activities-Lifespan* (ASD-DIAL; described in [28]), (2) *Adapted Virtual Autism Behavior Observation* (A-VABO; described in [28], (3) *Brief Observation of Symptoms of Autism* (BOSA; [43], 4) *Observation of Play Screener*: *Home Edition* (OOPS:HE; described in [28]), and TELE-ASD-PEDS [44]. All these approaches involve a clinician virtually observing and providing instructions as a parent engages in a series of semi-scripted activities with their child. The activities are specifically designed to elicit social behaviors and/or potential symptoms of ASD. Although these methods appear promising, only data from the TELE-ASD-PEDS has been officially published [44].

### Current review

The primary research question that guided this study was: *What evidence exists to support the use of telehealth methods to diagnose ASD*? A scoping review was conducted to provide an overview of the literature on the utilization of telehealth to either diagnose ASD or confirm an existing ASD diagnosis. Types of evidence to inform practice (i.e., outcomes of reliability, validity, and feasibility of telehealth from the perspectives of patients, caregivers, and/or diagnosticians), as well as characteristics of samples in included studies (e.g., sample race, socioeconomic status, type of community), were extracted to provide a better understanding of the successes and knowledge gaps in the literature. We also outline necessary adaptations and modifications to in-person procedures that are necessary for telehealth assessments. In line with the broad nature of scoping reviews [45], literature searches were not limited by participant age; samples of children, adolescents, and adults were all considered eligible. Ultimately, this scoping review may be a resource for researchers and practitioners, particularly those currently running telehealth programs or are planning to do so.

## Methods

**Fig 1:**
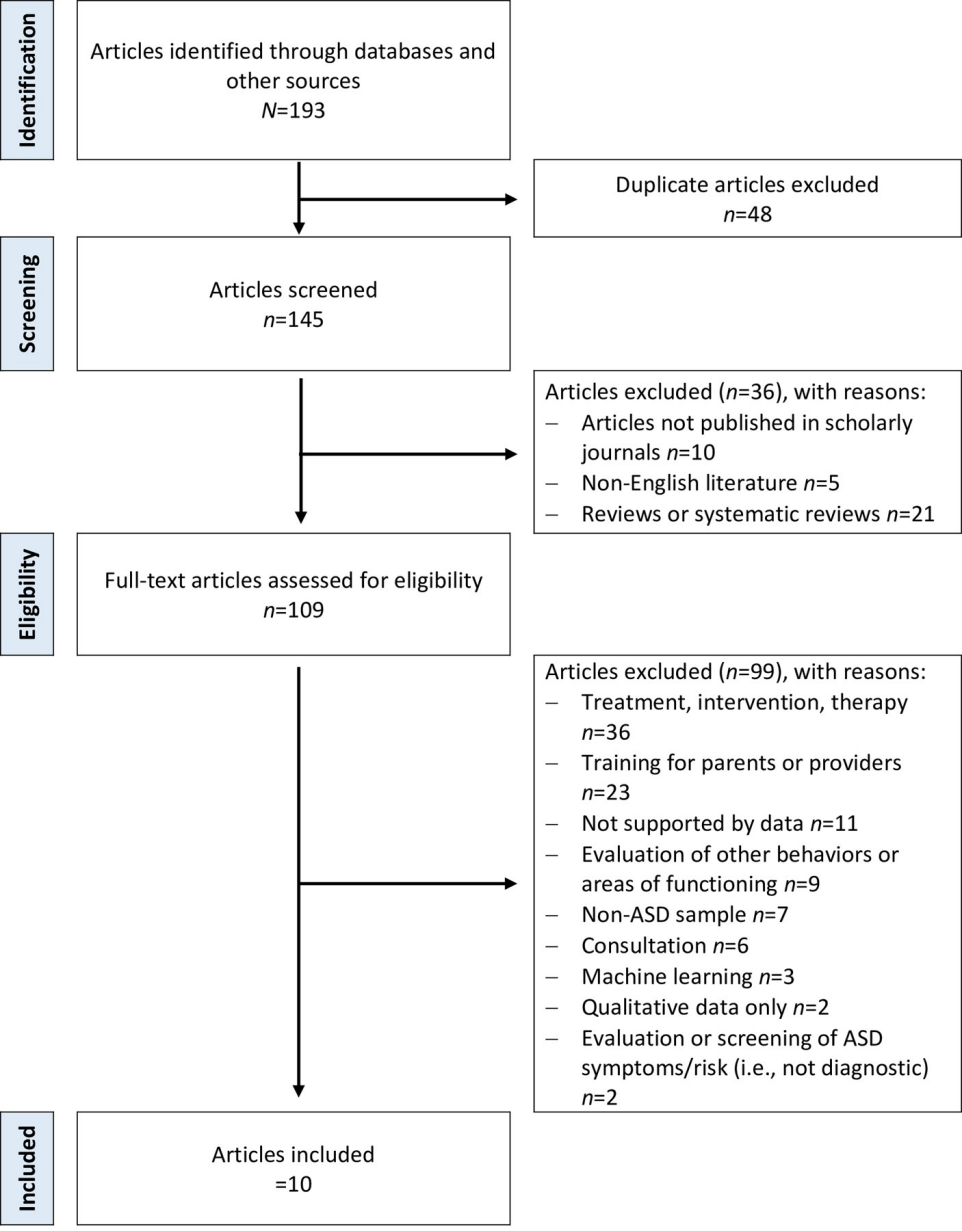
PRISMA flow diagram for selection of studies.

Given that reporting guidelines for scoping reviews are limited [46, 47], researchers have been advised to use the Preferred Reporting Items for Systematic Review and Meta-Analyses (PRISMA) framework [48]. As such, in this paper, PRISMA was utilized to guide procedures for identification, screening, eligibility, and inclusion of articles for review. Fig 1 provides an illustration of the selection process.

### Study search

PubMed and PsychInfo electronic databases were searched for published research pertaining to the use of telehealth for ASD diagnosis. Three parameters were utilized in the initial electronic search: ASD population, type of telehealth, and diagnostic procedures. The specific search terms are provided in Table 1. Two authors also searched the references lists of articles and systematic reviews identified by the specified search method. Literature searches were conducted through June 3^rd^, 2021.

**Table 1:** Search terms and reasons for exclusion.

**Search terms**	
Population of Interest	“ASPERGER” OR “PDD-NOS” OR “AUTISM” OR “AUTISTIC”
Type of telehealth	“TELEHEALTH” OR “TELEMEDICINE” OR “TELECARE” OR “VIDEOCONFERENCE*” OR “STORE AND FORWARD”
Diagnostic procedures	“DIAGNOSIS” OR “ASSESSMENT” OR “DIAGNOSTIC”
**Reasons for exclusion**	Not an original article (reviews/meta-analyses, proposals not supported by data)
	Not in English
	Not an ASD sample
	Evaluation of symptoms or behaviors not for diagnostic
	Assessment through machine learning or telephone only
	Training for parents or providers
	Treatment, intervention, or therapy
	Consultative or collaborative care
	Qualitative data only (e.g., stakeholder feedback only)

### Eligibility and data abstraction

To minimize bias in the identification of articles, the initial search was not filtered based on publication date, population, publication title, journal type, or article type [49]. Two authors manually reviewed the titles and abstracts of articles from the electronic search. Duplicate records were removed. Articles were excluded if only qualitative data were reported (e.g., case studies, descriptive data on social validity) in the absence of a telehealth diagnosis. In addition, exclusion criteria at the screening stage included source type, article type, and language. Specifically, articles were excluded if: they were not published in a scholarly/peer-reviewed journal; they were not original empirical studies (e.g., systematic reviews); or they were not published in English. Exclusion criteria are provided in Table 1. Reviewed articles were original research that reported outcomes of telehealth to diagnose ASD across all age groups.

Abstracted data included sample characteristics (Table 2), telehealth assessment information (Table 3), and information on outcomes of interest, specifically reliability, validity, social validity, and feasibility (Table 4). Data abstraction was completed by two authors separately. If the authors disagreed about data in a study, the study was discussed until agreement was reached. Given the variability in definitions of these concepts, outcomes of interest were assessed using the following definitions:

- Reliability: the extent to which results from telehealth diagnoses agreed with results from in-person assessments (e.g., percent agreement; accuracy).
- Validity (i.e., sensitivity and specificity): the extent to which telehealth assessments measured what they were designed to measure.
- Social validity: the extent to which telehealth was viewed as acceptable and important in the context of diagnostic services (e.g., feedback on the procedures by stakeholders).
- Feasibility: the extent to which telehealth can be realistically conducted and maintained (e.g., clinical feasibility as noted by providers; technology costs; cost savings; challenges associated with telehealth).

**Table 2:** Sample characteristics of included studies.

Study	Country	State/ Region	Sample	Age at evaluation	Sex	Race	SES	Type of community
Savin et al., 2006	USA	Rapid City, South Dakota and Denver, Colorado	21 children	*M* = 6.5 yrs.	NR	American Indian	**No sample data reported;** authors noted that the American Indian population is more economically disadvantaged than the general population	**No sample data reported;** authors noted that the American Indian population is more rural than the general population
Reese et al., 2013	USA	Midwest	21 children (11 with pre-existing diagnosis of ASD; 10 with pre-existing diagnosis of DD)	3–5 yrs.	18 males	19 white, 1 African American, 1 biracial,	NR	NR
Nazneen et al., 2015	USA	Georgia	5 children (4 with pre-existing diagnosis of ASD) 3 clinicians	2–6 yrs. (*M* = 4 yrs.)	NR	NR	NR	NR
Reese et al., 2015	USA	NR	17 families	2.4–5.8 yrs. (*M* = 4.47)	70.6% male	88.2% white, 5.9% African American 5.9% Hispanic	NR	NR
Schutte et al., 2015	USA	Pittsburg, Pennsylvania	23 adults with an existing diagnosis of an ASD	19–30 yrs. (*M* = 21.96, *SD* = 2.88)	16 males	NR	NR	**No sample data reported**; setting was identified as a rural clinic
Smith et al., 2017	USA	Southwest	51 families (11 TD children)	18 mos. - 6 yrs., 11 mos.	36 males	21 Caucasian, 22 Hispanic, 4 Black, 4 Other	NR	NR
Juarez et al., 2018	USA	Study 1: NR Study 2: Rural counties	Study 1: 20 families of children with early concerns about ASD Study 2: 45 families (29 children with ASD)	Study 1: 20–34 mos. (*M* = 26.65, *SD* = 4.49) Study 2: 19–32 mos. (*M* = 26.80, SD = 3.12)	Study 1: 16 males Study 2: 35 males	Study 1: NR Study 2: 30 White/Non-Hispanic, 9 Black/African American, 3 Hispanic, 3 Biracial	Study 1: NR Study 2: 62% of caregivers in the ASD sample reported highest grade completed as high school. Partnered with local regional center for families that had a high poverty rate; county data reported (median household income: $40,541.20; % living in poverty: 20.25)	Study 1: NR Study 2: No sample data reported; partnered with a regional center serving 23 rural county regions, geographically distant from the urban diagnostic centers in the state
Sutantio et al., 2020	Indonesia	Jakarta	40 families	18–30 mos.	29 males	NR	NR	NR
Wagner et al., 2020	USA	Tennessee, Alabama, Kentucky	204 children; 9 clinicians	16–36 mos. (*M* = 27.54; SD = 5.36)	157 males47 females	NR	NR	NR
Corona et al., 2021	USA	NR	51 families of young children (35 were recruited from a research database consisting of children with ASD and DD) 7 assessors	18–36 mos.	46 males 15 females	32 White 10 Black or African American 2 More than one race; 3 Hispanic or Latino	Within the ASD sample, 23% reported household income as $50,000 or less; 52% as $50,000-$100,000, 20% as $100,000 or more (6% did not answer)	NR

NOTE. If participants had a pre-existing diagnosis, the N was reported under the sample column. Information on SES (socioeconomic status) is provided in the table if parental education or income was reported in the study.

**Table 3:** Telehealth assessment information and procedures from included studies.

Study	Telehealth method and location	Telehealth assessment protocol and modifications	Telehealth assessment completion time	People involved in telehealth assessment procedures
Savin et al., 2006	Video-conferencing from home	Clinical evaluation	Approximately 80 minutes	Two general psychiatrists, one psychologist, and one nurse practitioner who have more than 20 collective years of full-time experience at the hospital; the two psychiatrists had more than 12 collective years of experience working with American Indians Both child and caregiver present during telehealth assessment.
Reese et al., 2013	Video-conferencing simulation at a university medical center; one room was connected through video-conferencing to an observation room in the same building	ADOS Module 1: Caregivers administered ADOS presses ADI-R: Only algorithm items were administered	NR	Five clinicians who were blinded to participant diagnosis; clinicians received training on the ADOS, ADI-R, and DSM-IV-TR diagnostic criteria for autism, and had experience working on interdisciplinary teams Caregiver facilitated the ADOS and was provided instructions on how to administer ADOS presses
Nazneen et al., 2015	Store and forward; home videos recorded and uploaded by caregivers	Clinical evaluation via NODA systems (i.e., smartCapture and Connect)	Using the NODA Connect portal, diagnosticians reported an average of 68 minutes to review videos and complete assessment; average of 37 minutes was reported for TD children	Three diagnosticians experienced in autism diagnosis and unfamiliar with diagnostic assessments via NODA systems Caregivers recorded as many as four, 10-minute long videos of the child in specific settings and uploaded them to the NODA assessment portal. Instructions were provided.
Reese et al., 2015	Video-conferencing simulation at a university medical center; one room was connected through video-conferencing to an observation room in the same building	Observation of unstructured play, modified ADOS-2 activities, ADI-R interview using algorithm items only, and medical/family history	NR	Four research clinicians worked in pairs (one per setting–videoconferencing and in-person) Caregivers were directed through modified ADOS-2 activities (following procedures from Reese et al., 2013)
Schutte et al., 2015	Video-conferencing at a clinician site (e.g., University of Pittsburgh) and a client site (e.g., rural clinic)	ADOS Module 4	NR	One Module 4 research reliable clinician Caregivers were not reported to be present or involved during telehealth assessments (likely due to the age of participants, i.e., young adults)
Smith et al., 2017	Store and forward; home videos recorded and uploaded by caregivers	Store and forward: Brief developmental history interview and DSM-5 checklist for ASD using video data In-person assessment: ADI-R, ADOS-2, VABS, MSEL/KBIT	Most completed the assessment in under an hour	Assessor: Psychologist with 20 years of experience evaluating individuals with ASD for research purposes Raters: Primary NODA rater with a master’s degree in psychology and 10 years conducting ASD assessments. 10 secondary raters, either clinical or research professionals, with a minimum of 10 years of experience conducting observational assessments for ASD; all raters received a 30-minute training on NODA procedures and assessment portal Caregivers recorded as many as four, 10-minute long videos of the child in specific settings and uploaded them to the NODA assessment portal. Instructions were provided, following procedures from Nazneen et al., 2015.
Juarez et al., 2018	Video-conferencing through clinic rooms at a health care facility	Study 1 and 2: STAT, DSM-5 Clinical Interview, DSM-5 Symptom Checklist, MSEL, VABS, ADOS-2	Telemedicine appointment lasted no more than 1-hour.	Study 1: licensed psychological provider Study 2: psychologists and STAT administrator Caregivers were not involved in the administration of assessments in Study 1 or 2.
Sutantio et al., 2020	Store and forward; home videos recorded and uploaded by caregivers	Indonesian-translated DSM-5 checklist for ASD	NR	Evaluation of videorecording was done by a psychologist with 15 years of experience diagnosing ASD. ASD diagnosis done by a pediatric neurologist with 30 years of experience. The two diagnosticians had 15 years of experience in the same clinic together.
Wagner et al., 2020	Video-conferencing from home using a personal device (smart phone, tablet, laptop) to access a Zoom video platform	TELE-ASD-PEDS	67% reported spending between 60 to 120 minutes and 33% reported spending 120–180 minutes during telemedicine visits	Nine licensed clinical psychology providers with expertise diagnosing ASD in young children (M years of experience in pediatric settings with children with ASD = 8 years, SD = 6.14 years, range 2–20 years). All clinicians were research reliable on the ADOS-2. Two clinicians reported using telemedicine in clinical practice. Caregivers were guided to complete interactive activities with their children during telehealth appointments.
Corona et al., 2021	Video-conferencing through tele-screening rooms (rooms connected by video conference technology to both the clinician and the family)	TELE-STAT, TELE-ASD-PEDS	On average, 23 minutes per tele-visit (SD = 5 minutes)	Seven licensed clinical psychologists and licensed senior psychological examiners with expertise in diagnosing ASD in young children. All assessors were research reliable on the ADOS-2. Caregivers were guided to complete interactive activities with their children during telehealth appointments.

**Table 4:** Outcomes of included studies.

Study	Reliability reported?	Validity reported?		Social Validity reported?	Feasibility reported?
		Sensitivity	Specificity		
Savin et al., 2006	No	No	No	Yes	Yes
Reese et al., 2013	Yes	No	No	Yes	Yes
Nazneen et al., 2015	Yes	Yes	Yes	Yes	Yes
Reese et al., 2015	Yes	Yes	Yes	No	No
Schutte et al., 2015	Yes	No	No	Yes	Yes
Smith et al., 2017	Yes	Yes	Yes	No	No
Juarez et al., 2018	Yes	Yes	No	Yes	Yes
Sutantio et al., 2020	Yes	Yes	Yes	No	No
Wagner et al., 2020	No	No	No	Yes	Yes
Corona et al., 2021	Yes	Yes	Yes	Yes	Yes

Due to the interconnectedness and overlap in the way that outcomes of reliability and validity were measured in the reviewed articles, we present these results together. Similarly, findings on social validity and feasibility are presented together in the sections below.

## Results

### Study selection

PubMed yielded 95 results in the initial search, and PsychInfo yielded 98 results, resulting in 193 total articles. After removing duplicate articles and screening abstracts and titles, 84 articles were excluded. By examining the remaining 109 articles in detail, 99 articles were further excluded. Thus, 10 articles were retained for this review (see Fig 1 and S1 Table). Notably, the majority of articles were excluded due to the exclusive focus on: treatment, intervention or therapy (*n* = 36); parent or provider training (*n* = 23); and evaluations of symptoms or behaviors not for diagnostic purposes (*n* = 11).

### Sample characteristics

Inclusionary criteria included language and requiring that manuscripts were published in English. Therefore, we note that nine of the 10 studies reported research conducted in the United States. One paper reported research conducted in Indonesia [50]. Among the studies included in this review, participants were largely male likely because ASD occurs more often in males than females at a ratio of approximately 4:1 [2]. Nearly all studies focused on young children (16 months– 6 years, 11 months); one study assessed adults (18–30 years). Four studies did not report participant race/ethnicity; of those that did, samples were predominantly identified as White or Caucasian. While the majority of included studies discussed telehealth as a viable method to bridge diagnostic disparities among vulnerable groups, particularly under-resourced or rural communities, only two studies provided data on their sample’s socioeconomic status (e.g., parent income, education) [34, 51]. None *systematically* reported on community types (e.g., rural, urban, suburban, etc.) of their sample, though one mentioned being in a rural area [52]. Study characteristics for all included studies are shown in Table 2.

### Telehealth assessment methods

Seven studies used videoconferencing, while three studies used store-and-forward methods. Of the seven studies that utilized videoconferencing, five described conducting on-site videoconferencing methods [34–36, 38, 51] for which two assessment rooms (e.g., at a clinic or university center) were connected by videoconferencing technology. These methods were primarily used to test and validate telehealth procedures prior to engaging in community studies. Four studies utilized variations of the “gold standard” assessments for ASD diagnosis (i.e., ADOS/ADOS-2 or ADI-R) as part of the assessment protocol; two studies utilized the TELE-ASD-PEDS [44, 51]. Two studies did not report on the length of time for telehealth assessments. Of the seven studies that reported the length of time for telehealth assessments, completion time (written as an average, approximation, or range) varied. For example, Corona et al. (2021) reported that telehealth visits were, on average, 23 minutes, whereas Wagner et al. (2020) reported that 33% of visits ranged from 120–180 minutes. All studies disclosed, to some degree, the amount of training or experience that clinicians or diagnosticians possessed. In studies where store-and-forward methods were utilized [31, 37, 50], caregivers were directly involved in recording and uploading home videos that trained assessors later scored for diagnostic purposes. Four studies involved caregivers by guiding them through the facilitation of interactive assessment activities via videoconferencing [36, 38, 44, 51]. Telehealth assessment information and procedures are displayed in Table 3.

### Reliability and validity

Table 4 indicates which studies included outcomes of reliability and validity. We operationally defined reliability as the percent agreement about diagnostic status (e.g., ASD or not ASD) for in-person and telehealth methods when assessed by blinded clinicians or clinical teams. For clarity, we use the term “accuracy” in this review when appropriate. When unblinded clinicians or clinical teams did telehealth and in-person methods, reliability is still discussed, but we note the possible confounds involved. When sufficient information was provided to calculate sensitivity and/or specificity (e.g., validity), values were reported.

Of the 10 studies reviewed, eight directly compared diagnostic accuracy between telehealth and in-person assessment methods for children with ASD (marked under “reliability” in Table 3). In total, the eight studies had a combined sample size of 228 comprised of individuals between 18 months and 30 years of age. However, only one study included participants older than 7 years of age (*n* = 23). Thus, of the total sample size of 228, 205 participants were between the ages of 18 months and 7 years, and the remaining 23 were between 19–30 years old. Diagnostic accuracy for telehealth assessment methods versus in-person ranged between 80% and 91%. However, Schutte and colleagues [35] compared accuracy on ADOS score (one of the “gold standard” assessments for ASD) rather than diagnostic accuracy itself. Thus, it was included in this section as the ADOS is utilized to diagnose ASD (among other measures). Savin and colleagues [52] did not report sufficient information about their assessment procedures, nor did they compare accuracy between telehealth vs. in-person assessments. Wagner et al. (2020) reported data on telehealth assessments only due to clinic closures from COVID-19 [44].

Regarding validity, six of the 10 studies provided sufficient information to calculate validity using sensitivity, specificity, or both (validity could not be calculated for [35, 36, 44, 52]. The six studies for which sensitivity could be calculated had a combined sample size of 184. Of these studies, sensitivity values were between 75% and 100%. For one study [34], sensitivity was calculated, but specificity was unable to be calculated due to the lack of a control group of children without ASD. Thus, specificity was calculated in five out of 10 studies, with a combined sample size of 164. Of the studies for which specificity was calculated, values were between 68.75% and 100%. These articles are summarized below.

In 2013, Reese and colleagues utilized video conferencing to administer both the *Autism Diagnostic Observation Schedule* (ADOS) and *Autism Diagnostic Interview-Revised* (ADI-R) to 21 children (aged 3–5 years) and parents [36]. Eleven children had a previous diagnosis of ASD, and ten had a diagnosis of developmental delay (DD). In this study, parents were instructed on completing social “presses” from the ADOS, while clinicians observed them either in person or via remote video (telehealth). Children were randomly assigned to complete the ADOS/ADI-R in-person or via telehealth. After the evaluations were completed, each assessor gave their diagnostic impression (e.g., ASD or DD), and diagnostic impressions were compared among assessors. The authors compared the scores from four assessors on both the ADOS and ADI-R between assessment settings (i.e., telehealth versus in-person). When comparing scores on individual ADOS items between clinicians in the telehealth versus in-person conditions, the average percent agreement was 71%, which was not significantly different from the percent agreement between clinicians in the same setting (72%). Similar results were observed for the ADI-R, with percent agreement at ~84% across settings and 87% when clinicians were in the same setting. Clinicians’ diagnostic impressions matched the child’s existing diagnosis 83% of the time in the in-person condition and 86% in the telehealth condition, with no significant differences observed between conditions. Information about sensitivity (e.g., true positive) and specificity (e.g., true negative) from diagnostic impressions between telehealth and existing diagnosis (e.g., DD versus ASD) was not provided and therefore cannot be reported here.

Nazneen and colleagues measured the accuracy of using home videos (recorded by parents) to diagnose ASD remotely [37]. Naturalistic Observation Diagnostic Assessment (NODA) smartCapture was utilized by parents to record clinically relevant videos of their child’s behavior, which were then forwarded to clinical providers. This procedure is referred to as store-and-forward. Parents of five children between 2–6 years of age (four with a previous diagnosis of ASD and one without) were given four scenarios to record: child playing alone, the child playing with a sibling or peer, family mealtime, and any other behavior that the parent deemed concerning. Caregivers were given specific instructions for eliciting social behavior from children (e.g., calling the child’s name to get his or her attention, pointing towards an object to see if the child would look at it). Two clinicians who were blind to the children’s previous diagnosis independently judged whether each child had ASD or not based on the videos and developmental history. For four of five children (three with a previous diagnosis of ASD and one who was neurotypical), both remote clinicians independently arrived at the same diagnostic conclusion, and those decisions were in agreement with the child’s previous diagnosis. In the case of the fifth child (with a previous diagnosis of ASD), one clinician’s decision matched the previous diagnosis, but the other did not. In this case, a third independent clinician reviewed all videos and developmental history and concluded that the child had ASD. Thus, out of 11 total assessments via NODA (two clinicians rated four of the children, and three clinicians rated the 5^th^ child as the first two clinicians did not agree), 10 reached a diagnostic conclusion that matched the child’s previous in-person diagnosis (91% accuracy). Regarding sensitivity and specificity, for one remote clinician, both sensitivity and specificity were 100%. For the second remote clinician, sensitivity was 75% (three out of four correctly identified as having ASD), and specificity was 100%.

Smith and colleagues also used NODA compared to an in-person evaluation with 51 children– 11 neurotypical children and 40 children for whom parents sought an ASD evaluation [31]. All participants were between 18 months and 6 years, 11 months of age. The primary NODA rater was blind to the child’s group membership (e.g., seeking an evaluation vs. neurotypical) and was blind to the results of the in-person assessment. The in-person evaluation included the ADOS-2, ADI-R, a cognitive assessment (either the *Mullen Scales of Early Learning* or the *Kaufman Brief Intelligence Test*), and the *Vineland Adaptive Behavior Scales*. Telehealth assessments involved both developmental history and video data collected with NODA. Across the 51 participants, raters of telehealth and in-person assessments agreed about the appropriate diagnosis (ASD or not ASD) in 88.2% of cases. In the subgroup of children for whom an evaluation was desired by parents (*n* = 40), the in-person and telehealth assessments resulted in agreement on diagnosis in 85% of cases. Sensitivity was 84.9% in both groups of children (n = 51). Specificity was 94% in the full sample and 85.7% in the subgroup of children whose parents desired an evaluation.

Reese and colleagues coached caregivers on completing a modified set of ADOS-2 activities with their child either in person or via video coaching, and had clinicians make diagnostic status judgments (e.g., ASD or no ASD) having watched the parent-child interaction either in-person or via video [38]. Ten families of children completed the in-person procedure, and seven completed the video procedure (total *n* = 17). Children were between 2.4 and 5.8 years old. Clinicians also completed the ADI-R algorithm items and read medical/family history information. Finally, all families were brought back to the clinic within 60 days for an in-person confirmatory assessment by blinded clinical teams. Clinicians observing the ADOS-2 activities in person were accurate in their diagnosis 82% of the time, compared to the clinical team who completed a hands-on blinded in-person assessment. Those observing via video were accurate 86% of the time compared to the in-person clinical team. Specificity was 78% for in-person observation and 88% for those observing via video; sensitivity was 88% for in-person observation and 84% for those observing via video.

In 2015, Schutte and colleagues compared telehealth versus in-person ADOS administration with 26 adolescents and adults with ASD (19–30 years of age) [35]. The authors utilized the Versatile and Integrated System for Telerehabilitation (VISYTER) platform, which has two cameras on the participant’s side (one static head-on view and a second remote-controlled camera). The system allowed stimuli to be seen by both the clinician and participant on a tablet, making it possible to engage in interactive activities involving a specific item. Half of the participants were randomly assigned to receive in-person testing first and subsequently complete testing via VISYTER, and the other half completed procedures in the opposite order. The two assessments were conducted at least 90 days apart to reduce learning or practice effects. Though the same clinician completed all assessments, a subset of randomly selected videos was scored by an outside “blinded” clinician. Agreement between the outside clinician and the assessor ranged from 82% (the reliability between clinicians on the ADOS algorithm) and 84.5% (the average agreement of individual ADOS items). To calculate reliability between assessment types (e.g., in-person versus VISYTER), correlation coefficients were utilized. The Intraclass correlation coefficient (ICC) on the ADOS classification (e.g., Autism, Autism Spectrum, Non-Spectrum) was .92. When individual ADOS domain scores were considered separately, the ICC was between .92-.98 (good) for “Communication”, “Social interaction,” and “Communication + Social interaction.” ICC was .70 (moderate) on the “Stereotyped Behaviors and Restricted Interest” domain score. Note that Schutte et al. (2015) did not explicitly measure *diagnostic* accuracy of telehealth versus in-person assessments, though they compared reliability on one of the two “gold standard” diagnostic assessments (ADOS) across telehealth and in-person administrations. Due to the study design, validity (sensitivity and specificity) could not be calculated.

Juarez and colleagues [34] measured the efficacy of a telehealth assessment procedure compared to a traditional in-person assessment in 20 children between 20–34 months of age. All children were referred for an ASD evaluation due to developmental concerns. The telehealth procedure included: a psychosocial interview, observation of the *Screening Tool for Autism in Toddlers and Young Children* (STAT; [53–55], and a DSM-5 ASD diagnostic interview. The psychosocial interview and DSM-5 interview were conducted by a clinician using telehealth, and the STAT was completed by a trained research assistant and observed by the same clinician using telehealth. Thus, part of the assessment procedure was completed in person, but the individual who conducted the in-person measure was not the clinician making a diagnostic judgment. Upon completion of the telehealth procedure, the clinician classified each child as having ASD or a different diagnosis (e.g., global developmental delay, language delay). The in-person assessment was completed by a blinded clinical psychologist and consisted of a cognitive assessment (the *Mullen Scales of Early Learning*; [56]), the *Vineland Adaptive Behavior Scales* (VABS [57], the *Autism Diagnostic Observation Schedule*, *Second Edition* (ADOS-2; [58], and the same DSM-5 clinical interview completed in the telehealth procedure.

Among the sample of 20 children, 15 were classified as having ASD via telehealth, and 19 were classified as having ASD via the in-person assessment. For one child, both telehealth and in-person clinicians diagnosed him or her with a developmental delay. Thus, there was diagnostic agreement between the telehealth and in-person clinicians for 16 out of 20 children. For the four for whom there was a disagreement between the telehealth and in-person clinicians, the telehealth clinician did not diagnose with ASD, whereas the in-person clinician diagnosed with ASD. Overall, the telehealth procedure resulted in a sensitivity of 78.95%. Specificity was unable to be calculated due to the lack of a “control” group of children without ASD.

In 2020, Sutantio and colleagues compared accuracy between a store-and-forward telehealth diagnostic assessment utilizing NODA and a traditional in-person assessment at a neurodevelopmental clinic in Indonesia [50]. Participants were 40 children between the ages of 18 and 30 months who were on the clinic waiting list due to concerns about speech, social skills, or both. Caregivers were instructed to create 2 to 5-minute video recordings of their child in three scenarios: playtime with others, playtime alone, and alarming behaviors. During the “playtime with others” scenario, caregivers were instructed to interact with the child in specific ways (e.g., calling the child’s name to get his or her attention, asking the child to share toys, pointing at something to direct the child’s attention, teasing the child by offering something but not giving it, and covering up the child’s toys such that he or she was unable to play with it). During both the “playtime alone” and “alarming behavior” scenarios, caregivers were told to record specific behaviors (e.g., repetitive non-speech vocalizations and/or “scripted” speech, hand flapping). Once the videos were forwarded, a clinician reviewed them completed the Indonesian translated DSM-5 checklist for ASD, and provided a diagnosis of either ASD or non-ASD based on the results.

Participants completed the in-person assessment within two weeks after forwarding videos, though parents were not provided any feedback until after the in-person assessment. The in-person assessment consisted of information about developmental history and direct observation of the child’s behavior. The DSM-5 checklist was used to make a diagnostic judgment. Agreement between the telehealth and in-person assessment diagnoses was 82.5% (*n* = 21). The telehealth assessment resulted in true-positive ASD diagnoses in 52.5% of children (*n* = 21), false-positive results for 12.5% (*n* = 5), true-negative results in 30% (*n* = 12), and false-negative results in 5% (*n* = 2). Overall, sensitivity of the telehealth diagnosis was 91.3%, positive predictive value was 80.7%, and specificity (negative predictive value) was 85.7%.

In 2021, Corona and colleagues reported initial data related to the feasibility, acceptability, and utility of the TELE-ASD-PEDS and TELE-STAT with 51 children between the ages of 18–36 months [51]. Of the 51 participants, 35 had a previous diagnosis of ASD, 10 had a previous diagnosis of developmental delay, and six were neurotypical. Participants were randomly selected to either receive the TELE-STAT (*n* = 24) or the TELE-ASD-PEDS (*n* = 27). The TELE-STAT was created by adapting the STAT (Stone et al., 2004; 2008) for telehealth screening of children between 18–36 months of age. The TELE-STAT includes 12 activities designed to elicit social behaviors in children (i.e., play, requesting, directing attention, and imitation). Similar to the TELE-ASD-PEDS, clinicians provided specific directions to parents about what activities to engage in and provided specific prompts for parents to complete with their children. For clinical agreement between previous diagnosis and telehealth, remote clinicians accurately identified 33 children as having ASD and 11 children who did not have ASD (i.e., were either neurotypical or previously diagnosed with a developmental delay). Five children previously diagnosed with developmental delays were incorrectly identified as having ASD by remote assessors, and two children with ASD were incorrectly identified as not having ASD by remote assessors. Taken together, diagnostic agreement between remote assessors and previous diagnoses was 86%. Overall, sensitivity was 94.2%, and specificity was 68.75%. Data on parent perceptions of telehealth methods are reported below.

### Social validity and feasibility

Table 3 indicates which studies included outcomes of social validity and feasibility. We operationally defined social validity as the extent to which stakeholders (e.g., parents, providers) viewed telehealth as acceptable and important in the context of diagnostic services. Feasibility was defined as the extent to which telehealth can be realistically conducted and maintained. As these constructs are closely related (e.g., some view feasibility as an aspect of social validity), they are covered together in this section. Seven of the reviewed articles provided outcome data on social validity and feasibility with a combined sample size of 390, see Table 3. These seven studies reported data from children, adolescents, and adults with ASD between the ages of 16 months and 30 years of age. As noted above, only one of the seven studies included participants older than 7 years (*n* = 23). Thus, of the 390 total participants, 367 were between 18 months and 7 years old.

Savin and colleagues assessed and/or consulted with families of 21 children using video conferencing [52]. Of the 21, three were noted to have ASD. While both adult patients and parents of child participants were apprehensive about telehealth initially, most felt comfortable with the technology by the end of the visit. When speaking with providers about their perceptions of telehealth visits, providers shared that rapport was more challenging to establish in telehealth than in-person visits. Minor technology difficulties were evident—the video and/or audio quality were interrupted (i.e., frozen image once per session; several-minute delay due to video connection). During these instances, providers transitioned to voice-only contact. Across patients, parents, and providers, the travel and cost estimates of telehealth were preferred to in-person visits.

In a study of 21 children (3–5 years old) and their parents, Reese and colleagues [36] examined whether there were differences in parent satisfaction between interactive video-conferencing and in-person conditions using a 7-point Likert scale survey. All parent participants completed seven questions on aspects of satisfaction. The authors then used all seven items to calculate an average score for overall satisfaction. There were no significant differences in mean satisfaction between conditions on any items. Due to the relatively small sample size, the authors also calculated effect sizes for additional interpretation. A moderate effect size was found on the single item for general satisfaction, where the mean score for the in-person condition (*M* = 7.00) was higher than for the video-conferencing condition (*M* = 6.82). Moreover, there was a moderate effect size using the average score from all seven items, such that the average score for the in-person condition (*M* = 6.57) was higher than for the video-conferencing condition (*M* = 6.23). The authors posited that larger sample sizes were needed to examine differences in satisfaction as having clinical significance. In order to detect a significant difference between conditions at .80 power, the authors would need a sample of 45 participants. Lastly, Reese et al. (2013) noted that some caregivers were not familiar with the social ‘presses’ that were requested. For example, some had difficulty setting up a situation that would elicit joint attention.

Nazneen and colleagues reported on the usability of NODA smartCapture from an in-field evaluation [37]. Data collection included parent ratings of usability, quality of recorded videos, and parents’ reliance on the help menu. Initial data from four parent participants prior to the in-field evaluation indicated an average usability score of 3 out of 5, and the number and length of recorded videos were inconsistent with instructions. Based on these results, three additional features were added to the NODA system: (1) four icons on the home screen showing the different required video scenarios, (2) clear cues indicating whether videos were being recorded, and (3) an auto-stop function ending the recording after 10 minutes. After these additions, four new parents rated the ease of use as 4 out of 5 points, and the five parents from the in-field evaluation submitted the correct number and length of videos. In addition, only two of the five families accessed the help menu, which suggests that the modifications reduced the need for technical support.

Using data from diagnosticians, Nazneen and colleagues [37] reported that the NODA system was appreciated as it allowed them to make a diagnosis based on naturalistic behaviors rather than those observed in a clinic. However, all diagnosticians noted that telehealth might not be a good fit for certain children (e.g., those under 2 years of age, those with subtle signs of ASD, those who are severely impaired). It was also noted that issues related to video quality could interfere with accuracy (e.g., poor lighting, poor video quality, and/or lack of an unobstructed view of the child).

In Schutte et al. (2015), 23 young adults with ASD (*M* = 21.96 years) completed a 6-item Post-ADOS Assessment User Satisfaction Questionnaire [35]. Overall, most participants felt comfortable using the technology and found the video and audio quality acceptable. Most participants *agreed* that the assessment captured a “true picture” of typical behavior and *disagreed* that there were things they were unable to say or do during the assessment. Most participants expressed being willing to do the assessment over the computer in the future. Fourteen participants received the remote administration after the in-person assessment. These participants were asked which condition was preferred: two participants preferred the remote system, five participants preferred the in-person administration, and seven had no preference. The authors suggested that ADOS administrators could benefit from training related to information technology, as troubleshooting was occasionally required (e.g., adjusting settings, securing internet connections).

Juarez and colleagues (2018) noted that participants utilized clinic rooms at a medical center (Study 1) and at a regional health center (Study 2) with cameras that had pan, tilt, and zoom functionality controlled by a remote assessor [34]. Assessors reported technical challenges related to audio and visual quality (e.g., low audio volume, video lag). Assessors more commonly reported these technical barriers in Study 1 compared to Study 2. The authors did not offer possible explanations for this, though it was noted that technical issues did not interfere with the evaluation process. When asked to provide suggestions for improving the telehealth appointment, two parent participants in Study 2 provided brief written comments about the technical challenges (i.e., “Sound”, “Fix video”). Only one other parent offered a comment (“Longer child evaluations”).

In 2020, Wagner and colleagues published findings using the TELE-ASD-PEDS and clinical interviews with 204 children (*N* = 157 male) between 16 and 36 months of age [44]. The TELE-ASD-PEDS is a novel tool developed to screen for ASD remotely. The TELE-ASD-PEDS was developed for minimally verbal or non-verbal children under 3 years of age. All children had been previously referred for an ASD evaluation. The TELE-ASD-PEDS evaluations were conducted by nine licensed psychologists who were reliable on the ADOS-2 and had expertise in diagnosing ASD in young children. After each evaluation, clinicians were asked to record their diagnostic impressions (e.g., ASD, no ASD, unsure), how confident they were in their diagnostic impressions, and whether further in-person testing was warranted. Clinicians were also asked to provide their feedback and impressions about the TELE-ASD-PEDS. Clinicians reported feeling comfortable completing assessments, making diagnoses, and providing recommendations via telehealth. Clinicians also provided written comments, expressing difficulties with technology (e.g., dropped calls, inconsistent audio, challenges with helping caregivers). Other comments pertained to home distractions (e.g., lack of access to play materials; other people in the room with caregiver and child), obtaining informed consent, reliance on caregivers to communicate observations (i.e., eye contact and language use), and differences in parents’ ability to understand task instruction. However, providers also identified observations in the home environment as a benefit and acknowledged benefits of telehealth for caregivers (e.g., eliminating barriers to travel).

In a study of the TELE-ASD-PEDS and TELE-STAT procedures with 51 children, Corona and colleagues [51] collected quantitative and qualitative survey data on parent perceptions and suggestions. Overall, survey data revealed that parents found telehealth assessments to be acceptable and comfortable, though 12% of families reported that telehealth did not elicit child behaviors of concern. Written feedback revealed parent concerns related to technology (e.g., audio quality, small size of the screen, children’s interest in touching the screen), screening activities (i.e., more or different activities), and amount of time for screening. With regards to the latter, the average screening lasted 23 minutes. While 19% of parents liked the shorter time of screening, 11% of parents suggested extending screening visits to 30–45 minutes. Compared to their child’s full diagnostic evaluations, 20% of parents expressed that the telehealth format was less personal. Moreover, in comparison to the full diagnostic evaluation, 44% of parents expressed liking the parent-led nature of the telehealth screening procedures, though 22% expressed that telehealth would be useful as a first step to an in-person evaluation.

## Discussion

Telehealth represents an alternative for those who have difficulties accessing in-person assessments or when in-person visits are not possible (e.g., the situation due to COVID-19). This scoping review explored the breadth of available evidence on telehealth methods for diagnosing ASD. Reports of accuracy and reliability indicate that telehealth is largely accurate as compared to in-person diagnosis (accuracy ranged between 80–91%) and has acceptable sensitivity (75–100%) and specificity (68.5–100%). Overall satisfaction ratings from parents and clinicians revealed acceptable social validity, and data indicate that telehealth is feasible though not without challenges. Below we summarize successes and knowledge gaps from the included studies in this review.

### Advantages of telehealth methods

Regarding the benefits and advantages of telehealth, studies reviewed herein described the following: flexibility afforded to the clinician (e.g., ability to watch video recordings submitted by parents in the evenings or on the weekends) [31], ability to see a child’s behavior in his or her ‘natural’ environment [37, 44], and cost savings associated with telehealth versus in-person visits [44, 52]. Additionally, parents reported being satisfied with telehealth procedures and enjoyed the parent-led nature of the assessment activities [51]. Similarly, in the study where adults received telehealth and in-person evaluations, participants reported being satisfied with telehealth procedures and were willing to complete telehealth assessments in the future [35]. Though telehealth procedures are not without challenges (see the subsequent section below), the studies reviewed here report largely positive impressions from both clinicians and parents.

### Assessment adaptations for telehealth

Some studies utilized video assessments conducted by an assessor [34, 35, 52], while others incorporated elements of video-coaching to allow various assessment activities to be completed “live” with a caregiver [36, 38, 44, 51]. Another group of studies utilized “store and forward” methods in which parents sent video clips of their child via a secure platform [31, 37, 50]. Regardless of the setting, the behaviors clinicians attended to were the same (e.g., potential restricted interests/repetitive behaviors, quality of social interactions/social communication). Thus, the setting appears to matter less than the actual behaviors observed, particularly from a clinical perspective. However, if researchers wish to combine findings from multiple studies or derive certain conclusions, diagnostic commonalities must be present to assure meaningful outcomes. For this reason, a set of telehealth guidelines would be useful to the field, such as those offered by Corona and colleagues for toddlers and very young children [51].

Telehealth assessment procedures generally require adaptations from business-as-usual in-person ASD assessments. For example, if a child with limited language were assessed in person with the ADOS-2, the protocol would include an imitation task during which the assessor engages in various actions with toys and then prompts the child to imitate those specific actions. In another ADOS-2 activity, the assessor blows bubbles using a fan-based bubble gun. Using telehealth, neither of these activities are feasible, as the assessor and child are not together in the same room. During telehealth, activities to elicit social behaviors (e.g., eye contact, smiling, pointing, gestures) would be completed either in the context of a parent video during a routine event or elicited by a parent during a naturalistic social interaction (e.g., rolling a ball or a toy car back and forth).

### Limitations and considerations

In this review, only studies in the published literature were included, potentially biasing results towards papers with significant findings. That is, some research on this topic might not be published due to null findings. It is also possible that including only studies published in English biased the current review towards findings from the Western world (i.e., from the USA, Canada, Australia, Great Britain). All but one of the studies included in the current review involved children (between 18 months and 7 years of age). It is unclear, then, whether telehealth is equally accurate, sensitive, and feasible for adolescents and adults, as there is significantly less evidence derived from older individuals. We also note that among studies included in the current review, most participants were Caucasian. Unfortunately, the lack of ethnic diversity in participant populations may be due to documented disparities in the age of initial screening/diagnosis of ASD among traditionally minoritized groups [2, 59].

Many studies reviewed reported technology issues [34, 35, 37, 44, 51, 52]. One recommendation put forward was to provide basic information technology (IT) training to clinicians [35] to equip them with solutions for common technology issues that may arise during a telehealth appointment. Another important consideration for using telehealth methods to diagnose ASD is that this modality is likely not appropriate for all children—particularly those who are extremely young or who display either subtle signs of ASD or are severely impaired [37]. Other researchers reported challenges communicating with parents about either task directions or how to set up specific play scenarios (36;44) along with challenges related to the home environment (e.g., other people in the room, the presence of distractions) [44].

### Future directions

While many community and private clinics continue to advertise the use of telehealth, it is important to examine empirical evidence supporting this diagnostic alternative. Given that telehealth is often promoted as a means to improve access to diagnostic services for under-resourced and rural communities, future studies should prioritize the collection and reporting of data that inform these assumptions, including sample socioeconomic status, ethnicity, and community type. As noted above, most studies included in the current review were published in the USA with Caucasian participants under 7 years of age. Future studies using telehealth should consciously include more age, gender, race, and ethnic diversity. An understanding of how such family and environmental characteristics influence outcomes of telehealth methods for ASD diagnosis would aid in the growing body of evidence on its usefulness and convenience for both families and providers.

Going forward, telehealth methods are likely to become more pervasive, particularly considering increasing costs of obtaining an evaluation (e.g., travel time, parents needing to take time off work to get children assessed, lack of available providers in rural areas) and barriers imposed by health concerns, such as pandemics. Professionals involved in screening and diagnosis for ASD should continue to validate new procedures; this is important so that screening centers do not become captive to one measure or set of procedures. Although the ADOS-2, as originally conceived, has stood as one of the two “gold standard” assessments for ASD, it is not always feasible to administer, particularly in community or school settings (as opposed to clinics or university settings). Barriers to administering the ADOS-2 still exist, including: an increasing population of children who are considered at-risk for ASD, a limited number of trained diagnosticians raising issues of fidelity in ADOS-2 administration, lack of equitable access to screening, particularly among underrepresented groups, and costs. Nonetheless, it is incontestable that telehealth has enabled autism diagnosis services, including the use of gold-standard instruments, to continue to fill a service need. With ever-improving technologies, it may not ultimately be THE answer; regardless, in all cases, the metrics of reliability, validity, and feasibility are paramount.

## Supporting information

S1 Checklist Preferred reporting items for systematic reviews and meta-analyses extension for scoping reviews (PRISMA-ScR) checklist. (DOCX)

S1 Table Studies excluded for review. (DOCX)
