# Supplementary material for: A scoping review of telehealth diagnosis of autism spectrum disorder
Source: PLoS One. 2022 Feb 10;17(2):e0263062. doi: 10.1371/journal.pone.0263062 (PMC8830614; doi:10.1371/journal.pone.0263062)
Supplement: S1 Table — (DOCX) [file pone.0263062.s002.docx]

**Table S1.**

*Studies excluded for review*

| **Number** | **Study** | **Reasons for exclusion** |
| --- | --- | --- |
|  | Abd-alrazaq et al., 2019 | Review |
|  | Adamou et al., 2021 | Consultative or collaborative care |
|  | Adams & Young, 2017 | Review |
|  | Ahamba et al., 2017 | Evaluation of other behaviors or areas of functioning |
|  | Alfuraydan et al., 2020 | Review |
|  | Alkhalifah & Aldhalaan, 2018 | Review |
|  | Alnemary et al., 2015 | Training for parents or providers |
|  | Alqahtani et al., 2021 | Not supported by data |
|  | Ameis et al., 2020 | Not supported by data |
|  | AACAP, 2017 | Not supported by data |
|  | Antezana et al., 2017 | Review |
|  | Ausenhus et al., 2019 | Training for parents or providers |
|  | Barretto et al., 2006 | Evaluation of other behaviors or areas of functioning |
|  | Bearss et al., 2018 | Training for parents or providers |
|  | Becevic et al., 2020 | Not supported by data |
|  | Benson et al., 2017 | Evaluation of other behaviors or areas of functioning |
|  | Berger et al., 2021 | Review |
|  | Bernie et al., 2021 | Training for parents or providers |
|  | Boisvert et al., 2010 | Review |
|  | Boisvert & Hall, 2014 | Review |
|  | Bosch et al., 2009 | Not in English |
|  | Bosch et al., 2017 | Not in English |
|  | Boutain et al., 2020 | Training for parents or providers |
|  | Buono et al., 2021 | Treatment, intervention, or therapy |
|  | Cameron et al., 2020 | Treatment, intervention, or therapy |
|  | Clarke, 2018 | Not supported by data |
|  | Clifford et al., 2007 | Evaluation of symptoms or behaviors not for diagnostic of ASD symptoms/risk |
|  | Conaughton et al., 2017 | Treatment, intervention, or therapy |
|  | Conti et al., 2020 | Not supported by data |
|  | Cox et al., 2020 | Not supported by data |
|  | Craig et al., 2021 | Training for parents or providers |
|  | D’Agostino et al., 2020 | Training for parents or providers |
|  | Dahiya et al., 2021 | Review |
|  | Dimitropoulos et al., 2017 | Treatment, intervention, or therapy |
|  | Doyen et al., 2018 | Review |
|  | Doyen et al., 2019 | Not in English |
|  | Dunleavy et al., 2013 | Treatment, intervention, or therapy |
|  | Dykens, 2015 | Review |
|  | Fletcher et al., 2010 | Not supported by data |
|  | Gatti et al., 2007 | Evaluation of other behaviors or areas of functioning |
|  | Gerow et al., 2021 | Treatment, intervention, or therapy |
|  | Gettings et al., 2015 | Treatment, intervention, or therapy |
|  | Goldstein & Glueck, 2016 | Treatment, intervention, or therapy |
|  | Goldstein et al., 2017 | Review |
|  | Hao et al., 2021 | Treatment, intervention, or therapy |
|  | Heitzman-Powell et al., 2014 | Training for parents or providers |
|  | Hepburn et al., 2016 | Treatment, intervention, or therapy |
|  | Hilty et al., 2000 | Consultative or collaborative care |
|  | Hollis et al., 2016 | Review |
|  | Humm et al., 2014 | Treatment, intervention, or therapy |
|  | Ingersoll & Berger, 2015 | Training for parents or providers |
|  | Ingersoll et al., 2017 | Training for parents or providers |
|  | Jacques et al., 2018 | Treatment, intervention, or therapy |
|  | Jeste et al., 2020 | Treatment, intervention, or therapy |
|  | Johnsson et al., 2019 | Treatment, intervention, or therapy |
|  | Jung et al., 2006 | Treatment, intervention, or therapy |
|  | Jurek et al., 2021 | Treatment, intervention, or therapy |
|  | Kelleher et al., 2020 | Not an ASD sample |
|  | Khandaker, 2009 | Treatment, intervention, or therapy |
|  | Kim & Clark, 2015 | Treatment, intervention, or therapy |
|  | Knutsen et al., 2016 | Review |
|  | Kobak et al., 2011 | Training for parents or providers |
|  | Krysta et al., 2017 | Treatment, intervention, or therapy |
|  | Kuravackel et al., 2018 | Training for parents or providers |
|  | Lee et al., 2014 | Training for parents or providers |
|  | Lesser et al., 2019 | Evaluation of other behaviors or areas of functioning |
|  | Lindgren et al., 2020 | Training for parents or providers |
|  | Little et al., 2018 | Treatment, intervention, or therapy |
|  | Loman et al., 2021 | Not supported by data |
|  | Machalicek et al., 2009 | Evaluation of other behaviors or areas of functioning |
|  | Machalicek et al., 2016 | Consultative or collaborative care |
|  | Marcus et al., 2017 | Treatment, intervention, or therapy |
|  | Marino et al., 2020 | Treatment, intervention, or therapy |
|  | Martino & Hedderly, 2019 | Review |
|  | Max & Burke, 1997 | Treatment, intervention, or therapy |
|  | Mazurek et al., 2020 | Training for parents or providers |
|  | McCarty et al., 2015 | Not an ASD sample |
|  | McDuffie et al., 2016 | Not an ASD sample |
|  | Miyahara et al., 2009 | Treatment, intervention, or therapy |
|  | Narzisi, 2020 | Not supported by data |
|  | Nazneen et al., 2015 | Qualitative data only |
|  | Neely et al., 2017 | Treatment, intervention, or therapy |
|  | Oberleitner et al., 2004 | Treatment, intervention, or therapy |
|  | Oberleitner et al., 2007 | Treatment, intervention, or therapy |
|  | Parmanto et al., 2013 | Qualitative data only |
|  | Parsons et al., 2017 | Review |
|  | Pearl et al., 2014 | Consultative or collaborative care |
|  | Peterson et al., 2021 | Not an ASD sample |
|  | Pickard et al., 2016 | Treatment, intervention, or therapy |
|  | Pidano et al., 2016 | Consultative or collaborative care |
|  | Raman et al., 2019 | Evaluation of other behaviors or areas of functioning |
|  | Raspa et al., 2018 | Not an ASD sample |
|  | Reese et al., 2015 | Training for parents or providers |
|  | Rockhill et al., 2013 | Not an ASD sample |
|  | Saint-André et al., 2011 | Not in English |
|  | Salomone et al., 2017 | Training for parents or providers |
|  | Sehlin et al., 2018 | Treatment, intervention, or therapy |
|  | Shah et al., 2019 | Training for parents or providers |
|  | Shire et a., 2020 | Training for parents or providers |
|  | Simacek et al., 2017 | Treatment, intervention, or therapy |
|  | Sivaraman et al., 2021 | Treatment, intervention, or therapy |
|  | Sohl et al., 2017 | Training for parents or providers |
|  | Stainbrook et al., 2019 | Consultative or coordinated care |
|  | Stiles-Shields et al., 2020 | Review |
|  | Storey, 2010 | Not supported by data |
|  | Stuckey et al., 2017 | Review |
|  | Sutherland et al., 2018 | Review |
|  | Sutherland et al., 2019 | Evaluation of other behaviors or areas of functioning |
|  | Szeftel et al., 2012 | Evaluation of other behaviors or areas of functioning |
|  | Talbot et al., 2020 | Evaluation of symptoms or behaviors not for diagnostic of ASD symptoms/risk |
|  | Tan-MacNeill et al., 2020 | Treatment, intervention, or therapy |
|  | Tang et al., 2021 | Treatment, intervention, or therapy |
|  | Tariq et al., 2018 | Assessment through machine learning or telephone only |
|  | Tariq et al., 2019 | Assessment through machine learning or telephone only |
|  | Terry, 2009 | Treatment, intervention, or therapy |
|  | Tichon et al., 2003 | Treatment, intervention, or therapy |
|  | Todorow et al., 2018 | Review |
|  | Tsami et al., 2019 | Training for parents or providers |
|  | Valentine et al., 2021 | Review |
|  | van der Gaag, 2019 | Not in English |
|  | Vismara et al., 2013 | Training for parents or providers |
|  | Wacker et al., 2013 | Evaluation of other symptoms or areas of functioning |
|  | Washington et al., 2021 | Assessment through machine learning or telephone only |
|  | Wittingham & Coons-Harding, 2021 | Not an ASD sample |
|  | Wiederhold & Wiederhold, 2004 | Treatment, intervention, or therapy |
|  | Xie et al., 2013 | Training for parents or providers |

1. Abd-alrazaq, A.A., Alajlani, M., Alalwan, A.A., Bewick, B.M., Gardner, P., & Househ, M. (2019). An overview of the features of chatbots in mental health: A scoping review. *International Journal of Medical Informatics, 132*, 103978. <https://doi.org/10.1016/j.ijmedinf.2019.103978>
2. Adamou, M., Jones, S. L., Fullen, T., Galab, N., Abbott, K., & Yasmeen, S. (2021). Remote assessment in adults with Autism or ADHD: A service user satisfaction survey. *PloS one*, *16*(3), e0249237. https://doi.org/10.1371/journal.pone.0249237
3. Ahamba, G., Roberts, D., & Eachus, P. (2017). Triggering and measuring social inhibitory response in humans immersed in interactions with virtual humans.*Annual Review of CyberTherapy and Telemedicine, 15*, 185-188. Retrieved from https://search.proquest.com/scholarly-journals/triggering-measuring-social-inhibitory-response/docview/2027387679/se-2?accountid=14521
4. Adams...
5. Alfuraydan, M., Croxall, J., Hurt, L., Kerr, M., & Brophy, S. (2020). Use of telehealth for facilitating the diagnostic assessment of Autism Spectrum Disorder (ASD): A scoping review.*PLoS ONE, 15*(7), 16. http://dx.doi.org/10.1371/journal.pone.0236415
6. Alkhalifah, S., & Aldhalaan, H. (2018). Telehealth services for children with autism spectrum disorders in rural areas of the kingdom of Saudi Arabia: Overview and recommendations. *JMIR pediatrics and parenting*, *1*(2), e11402. https://doi.org/10.2196/11402
7. Alnemary, F. M., Wallace, M., Symon, J. B. G., & Barry, L. M. (2015). Using international videoconferencing to provide staff training on functional behavioral assessment.*Behavioral Interventions, 30*(1), 73-86. http://dx.doi.org/10.1002/bin.1403
8. Alqahtani, M., Alkhamees, H. A., Alkhalaf, A. M., Alarjan, S. S., Alzahrani, H. S., AlSaad, G. F., Alhrbi, F. H., Wahass, S. H., Khayat, A. H., & Alqahtani, K. (2021). Toward establishing telepsychology guideline. Turning the challenges of COVID-19 into opportunity. *Ethics, medicine, and public health*, *16*, 100612. https://doi.org/10.1016/j.jemep.2020.100612
9. Ameis, S. H., Lai, M. C., Mulsant, B. H., & Szatmari, P. (2020). Coping, fostering resilience, and driving care innovation for autistic people and their families during the COVID-19 pandemic and beyond. Molecular autism, 11(1), 61. https://doi.org/10.1186/s13229-020-00365-y
10. American Academy of Child and Adolescent Psychiatry (AACAP) Committee on Telepsychiatry and AACAP Committee on Quality Issues (2017). Clinical update: Telepsychiatry with children and adolescents. *Journal of the American Academy of Child and Adolescent Psychiatry*, *56*(10), 875–893. https://doi.org/10.1016/j.jaac.2017.07.008
11. Antezana, L., Scarpa, A., Valdespino, A., Albright, J., & Richey, J.A. (2017). Rural trends in diagnosis and services for autism spectrum disorder. *Frontiers in Psychology.* <https://doi.org/10.3389/fpsyg.2017.00590>
12. Ausenhus, J. A., & Higgins, W. J. (2019). An evaluation of real-time feedback delivered via telehealth: Training staff to conduct preference assessments.*Behavior Analysis in Practice, 12*(3), 643-648. http://dx.doi.org/10.1007/s40617-018-00326-1
13. Barretto, A., Wacker, D. P., Harding, J., Lee, J., & Berg, W. K. (2006). Using telemedicine to conduct behavioral assessments.*Journal of Applied Behavior Analysis, 39*(3), 333-340. http://dx.doi.org/10.1901/jaba.2006.173-04
14. Bearss, K., Burrell, T. L., Challa, S. A., Postorino, V., Gillespie, S. E., Crooks, C., & Scahill, L. (2018). Feasibility of parent training via Telehealth for children with autism spectrum disorder and disruptive behavior: A demonstration pilot.*Journal of Autism and Developmental Disorders, 48*(4), 1020-1030. http://dx.doi.org/10.1007/s10803-017-3363-2
15. Becevic, M., Wallach, E., Sheets, L. R., Misterovich, H., Norris, S., Aboagye, E., Dyer, J. A., Bacon, B. R., Edison, K., & Sohl, K. (2020). Accelerating care through ECHO: Case examples from the field. *Missouri medicine*, *117*(3), 235–240.
16. Benson, S. S., Dimian, A. F., Elmquist, M., Simacek, J., McComas, J. J., & Symons, F. J. (2018). Coaching parents to assess and treat self-injurious behaviour via telehealth. *Journal of Intellectual Disability Research: JIDR*, *62*(12), 1114–1123. https://doi.org/10.1111/jir.12456
17. Berger, N. I., Wainer, A. L., Kuhn, J., Bearss, K., Attar, S., Carter, A. S., Ibanez, L. V., Ingersoll, B. R., Neiderman, H., Scott, S., & Stone, W. L. (2021). Characterizing available tools for synchronous virtual assessment of toddlers with suspected autism spectrum disorder: A brief report. *Journal of Autism and Developmental Disorders*, 1–12. Advance online publication. https://doi.org/10.1007/s10803-021-04911-2
18. Bernie, C., Williams, K., Graham, F., & May, T. (2021). Coaching while waiting for autism spectrum disorder assessment: Protocol of a pilot feasibility study for a randomized controlled trial on occupational performance coaching and service navigation support. *JMIR research protocols*, *10*(1), e20011. https://doi.org/10.2196/20011
19. Boisvert, M., Lang, R., Andrianopoulos, M., & Boscardin, M. L. (2010). Telepractice in the assessment and treatment of individuals with autism spectrum disorders: A systematic review.*Developmental Neurorehabilitation, 13*(6), 423-432. http://dx.doi.org/10.3109/17518423.2010.499889
20. Boisvert, M., & Hall, N. (2014). The use of telehealth in early autism training for parents: A scoping review. *Smart Homecare Technology and TeleHealth*, *2*, 19-27. https://doi.org/10.2147/SHTT.S45353
21. Bosch, R. F., Krause-Allmendinger, H., Villena, P., & Kruck, I. (2009). Telemonitoring implantierbarer Aggregate. Praktische Aspekte und laufende Studien [Telemonitoring of implantable devices. Practical aspects and ongoing studies]. *Herzschrittmachertherapie & Elektrophysiologie*, *20*(4), 179–184. https://doi.org/10.1007/s00399-009-0059-0
22. Bosch, R., & Mutscher, I. (2017). Telemetrische Nachsorge von implantierbaren kardialen Aggregaten : Optimierung der Betreuung in der Praxis [Telemetric follow-up of implantable electronic cardiac devices : Optimisation of care in clinical practice]. *Herzschrittmachertherapie & Elektrophysiologie*, *28*(3), 260–267. https://doi.org/10.1007/s00399-017-0522-2
23. Boutain, A. R., Sheldon, J. B., & Sherman, J. A. (2020). Evaluation of a telehealth parent training program in teaching self‐care skills to children with autism.*Journal of Applied Behavior Analysis, 53*(3), 1259-1275. http://dx.doi.org/10.1002/jaba.743
24. Buono, S., Zingale, M., Città, S., Mongelli, V., Trubia, G., Mascali, G., Occhipinti, P., Pettinato, E., Ferri, R., Gagliano, C., & Greco, D. (2021). Clinical management of individuals with Intellectual Disability: The outbreak of Covid-19 pandemic as experienced in a clinical and research center Research in Developmental Disabilities. *Research in developmental disabilities*, *110*, 103856. https://doi.org/10.1016/j.ridd.2021.103856
25. Cameron, M. J., Moore, T., Bogran, C., & Leidt, A. (2020). Telehealth for family guidance: Acceptance and commitment therapy, parent-focused preference assessment, and activity-based instruction for the support of children with autism spectrum disorder and their families.*Behavior Analysis in Practice,*http://dx.doi.org/10.1007/s40617-020-00443-w
26. Clarke, C. S. (2018). Telepsychiatry in Asperger’s syndrome.*Irish Journal of Psychological Medicine, 35*(4), 325-328. http://dx.doi.org/10.1017/ipm.2017.19
27. Clifford, S., Young, R. & Williamson, P. (2007). Assessing the Early Characteristics of Autistic Disorder using Video Analysis. *Journal of Autism and Developmental Disorders.* 37**,**301–313. https://doi.org/10.1007/s10803-006-0160-8
28. Conaughton, R. J., Donovan, C. L., & March, S. (2017). Efficacy of an internet-based CBT program for children with comorbid High Functioning Autism Spectrum Disorder and anxiety: A randomised controlled trial. *Journal of affective disorders*, *218*, 260–268. https://doi.org/10.1016/j.jad.2017.04.032
29. Conti, E., Chericoni, N., Costanzo, V., Lasala, R., Mancini, A., Prosperi, M., Tancredi, R., Muratori, F., Calderoni, S., & Apicella, F. (2020). Moving toward telehealth surveillance services for toddlers at risk for autism during the COVID-19 pandemic. *Frontiers in psychiatry*, *11*, 565999. https://doi.org/10.3389/fpsyt.2020.565999
30. Cox, D. J., Plavnick, J. B., & Brodhead, M. T. (2020). A Proposed Process for Risk Mitigation During the COVID-19 Pandemic. *Behavior analysis in practice*, *13*(2), 1–7. Advance online publication. https://doi.org/10.1007/s40617-020-00430-1
31. Craig, E., Dounavi, K., & Ferguson, J. (2021). Telehealth to train interventionists teaching functional living skills to children with autism spectrum disorder.*Journal of Applied Behavior Analysis,*http://dx.doi.org/10.1002/jaba.834
32. D'Agostino, S., Douglas, S. N., & Horton, E. (2020). Inclusive preschool practitioners’ implementation of naturalistic developmental behavioral intervention using telehealth training.*Journal of Autism and Developmental Disorders, 50*(3), 864-880. http://dx.doi.org/10.1007/s10803-019-04319-z
33. Dahiya, A. V., DeLucia, E., McDonnell, C. G., & Scarpa, A. (2021). A systematic review of technological approaches for autism spectrum disorder assessment in children: Implications for the COVID-19 pandemic. *Research in developmental disabilities*, *109*, 103852. https://doi.org/10.1016/j.ridd.2021.103852
34. Dimitropoulos, A., Zyga, O., & Russ, S. (2017). Evaluating the Feasibility of a Play-Based Telehealth Intervention Program for Children with Prader-Willi Syndrome. *Journal of autism and developmental disorders*, *47*(9), 2814–2825. https://doi.org/10.1007/s10803-017-3196-z
35. Doyen, C. M., Oreve, M.-J., Desailly, E., Goupil, V., Zarca, K., L’Hermitte, Y., Chaste, P., Bau, M.-O., Beaujard, D., Haddadi, S., Bibay, A., Contejean, Y., Coutrot, M.-T., Crespin, L., Frioux, I., Speranza, M., Francois, N., & Kaye, K. (2018). Telepsychiatry for Children and Adolescents: A Review of the PROMETTED Project. *Telemedicine and E-Health*, *24*(1), 3–10. https://doi.org/10.1089/tmj.2017.0041
36. Doyen, C., Goupil, V., Desailly, E., Oreve, M. J., & Kaye, K. (2019). Télémédecine et troubles du spectre de l’autisme de l’enfant et de l’adolescent: Guide théorique et pratique.*Annales Médico-Psychologiques, 177*(7), 702-709. http://dx.doi.org/10.1016/j.amp.2019.04.017
37. Dunleavy, L., Preissner, K. L., & Finlayson, M. L. (2013). Facilitating a teleconference-delivered fatigue management program: Perspectives of occupational therapists.*Canadian Journal of Occupational Therapy / Revue Canadienne D'Ergothérapie, 80*(5), 304-313. http://dx.doi.org/10.1177/0008417413511787
38. Dykens, E. M. (2015). Family adjustment and interventions in neurodevelopmental disorders.*Current Opinion in Psychiatry, 28*(2), 121-126. https://search.proquest.com/scholarly-journals/family-adjustment-interventions/docview/1662001641/se-2?accountid=14521
39. Fletcher, R. R., Poh, M. Z., & Eydgahi, H. (2010). Wearable sensors: opportunities and challenges for low-cost health care. *Annual International Conference of the IEEE Engineering in Medicine and Biology Society. IEEE Engineering in Medicine and Biology Society. Annual International Conference*, *2010*, 1763–1766. https://doi.org/10.1109/IEMBS.2010.5626734
40. Gatti, E., Gatti, R. C., Lops, T., Massari, R., Sacchelli, C., & Riva, G. (2007). Virtual reality protocol: An instrument to assess alcohol-dependent individuals.*Annual Review of CyberTherapy and Telemedicine, 5*, 53-61. https://search.proquest.com/scholarly-journals/virtual-reality-protocol-instrument-assess/docview/622187811/se-2?accountid=14521
41. Gerow, S., Radhakrishnan, S., Davis, T. N., Zambrano, J., Avery, S., Cosottile, D. W., & Exline, E. (2021). Parent‐implemented brief functional analysis and treatment with coaching via telehealth.*Journal of Applied Behavior Analysis, 54*(1), 54-69. http://dx.doi.org/10.1002/jaba.801
42. Gettings, S., Franco, F., & Santosh, P. J. (2015). Facilitating support groups for siblings of children with neurodevelopmental disorders using audio-conferencing: A longitudinal feasibility study.*Child and Adolescent Psychiatry and Mental Health, 9*, 15. http://dx.doi.org/10.1186/s13034-015-0041-z
43. Goldstein, F., & Glueck, D. (2016). Developing Rapport and Therapeutic Alliance During Telemental Health Sessions with Children and Adolescents. *Journal of Child and Adolescent Psychopharmacology*, *26*(3), 204–211. https://doi.org/10.1089/cap.2015.0022
44. Goldstein, F.P., Klaiman, C., & Willliams, S. (2017). Bridging care gaps: Using tele-health to provide care for people with autism spectrum disorder. *International Journal of Developmental Disabilities, 63*(4), 190-194. DOI: [10.1080/20473869.2017.1322342](https://doi.org/10.1080/20473869.2017.1322342)
45. Hao, Y., Franco, J. H., Sundarrajan, M., & Chen, Y. (2021). A Pilot Study Comparing Tele-therapy and In-Person Therapy: Perspectives from Parent-Mediated Intervention for Children with Autism Spectrum Disorders. *Journal of autism and developmental disorders*, *51*(1), 129–143. https://doi.org/10.1007/s10803-020-04439-x
46. Heitzman-Powell, L., Buzhardt, J., Rusinko, L. C., & Miller, T. M. (2014). Formative evaluation of an ABA outreach training program for parents of children with autism in remote areas.*Focus on Autism and Other Developmental Disabilities, 29*(1), 23-38. http://dx.doi.org/10.1177/1088357613504992
47. Hepburn, S. L., Blakeley-Smith, A., Wolff, B., & Reaven, J. A. (2016). Telehealth delivery of cognitive-behavioral intervention to youth with autism spectrum disorder and anxiety: A pilot study.*Autism, 20*(2), 207-218. http://dx.doi.org/10.1177/1362361315575164
48. Hilty, D. M., Sison, J. I., Nesbitt, T. S., & Hales, R. E. (2000). Telepsychiatric consultation for ADHD in the primary care setting.*Journal of the American Academy of Child & Adolescent Psychiatry, 39*(1), 15-16. http://dx.doi.org/10.1097/00004583-200001000-00009
49. Hollis, C., Falconer, C. J., Martin, J. L., Whittington, C., Stockton, S., Glazebrook, C., & Davies, E. B. (2017). Annual Research Review: Digital health interventions for children and young people with mental health problems - a systematic and meta-review. *Journal of child psychology and psychiatry, and allied disciplines*, *58*(4), 474–503. https://doi.org/10.1111/jcpp.12663
50. Humm, L. B., Olsen, D., Be, M., Fleming, M., & Smith, M. (2014). Simulated job interview improves skills for adults with serious mental illnesses.*Annual Review of CyberTherapy and Telemedicine, 12*, 50-54. https://search.proquest.com/scholarly-journals/simulated-job-interview-improves-skills-adults/docview/1650985274/se-2?accountid=14521
51. Ingersoll, B., & Berger, N. I. (2015). Parent engagement with a telehealth-based parent-mediated intervention program for children with autism spectrum disorders: Predictors of program use and parent outcomes.*Journal of Medical Internet Research, 17*(10)http://dx.doi.org/10.2196/jmir.4913
52. Ingersoll, B., Shannon, K., Berger, N., Pickard, K., & Holtz, B. (2017). Self-directed telehealth parent-mediated intervention for children with autism spectrum disorder: Examination of the potential reach and utilization in community settings.*Journal of Medical Internet Research, 19*(7), 410-420. http://dx.doi.org/10.2196/jmir.7484
53. Jacques, C., Cloutier, V., & Bouchard, S. (2018). The “Decoding of social interactions in virtual reality” tasks for autism spectrum people: Development of an intervention protocol and pilot testing.*Annual Review of CyberTherapy and Telemedicine, 16*, 148-152. https://search.proquest.com/scholarly-journals/decoding-social-interactions-virtual-reality/docview/2321838904/se-2?accountid=14521
54. Jeste, S., Hyde, C., Distefano, C., Halladay, A., Ray, S., Porath, M., Wilson, R. B., & Thurm, A. (2020). Changes in access to educational and healthcare services for individuals with intellectual and developmental disabilities during covid‐19 restrictions.*Journal of Intellectual Disability Research,*http://dx.doi.org/10.1111/jir.12776
55. Johnsson, G., Kerslake, R., & Crook, S. (2019). Delivering allied health services to regional and remote participants on the autism spectrum via video-conferencing technology: lessons learned. *Rural and remote health*, *19*(3), 5358. https://doi.org/10.22605/RRH5358
56. Jung, K. -., Lee, H. -., Lee, Y. -., & Lee, J. -. (2006). Efficacy of sensory integration treatment based on virtual reality - tangible interaction for children with autism.*Annual Review of CyberTherapy and Telemedicine, 4*, 45-49. https://search.proquest.com/scholarly-journals/efficacy-sensory-integration-treatment-based-on/docview/622187875/se-2?accountid=14521
57. Jurek, L., Occelli, P., Denis, A., Amestoy, A., Maffre, T., Dauchez, T., Oreve, M. J., Baghdadli, A., Schroder, C., Jay, A., Zelmar, A., Revah-Levy, A., Gallifet, N., Aldred, C., Garg, S., Green, J., Touzet, S., Geoffray, M. M., & IFPAD study group (2021). Efficacy of parent-mediated communication-focused treatment in toddlers with autism (PACT) delivered via videoconferencing: a randomised controlled trial study protocol. *BMJ open*, *11*(4), e044669. https://doi.org/10.1136/bmjopen-2020-044669
58. Kelleher, B. L., Halligan, T., Witthuhn, N., Neo, W. S., Hamrick, L., & Abbeduto, L. (2020). Bringing the Laboratory Home: PANDABox Telehealth-Based Assessment of Neurodevelopmental Risk in Children. *Frontiers in psychology*, *11*, 1634. https://doi.org/10.3389/fpsyg.2020.01634
59. Khandaker, M. (2009). Designing affective video games to support the social-emotional development of teenagers with autism spectrum disorders.*Annual Review of CyberTherapy and Telemedicine, 7*, 37-39. https://search.proquest.com/scholarly-journals/designing-affective-video-games-support-social/docview/754032301/se-2?accountid=14521
60. Kim, S., & Clarke, E. (2015). Case study: An iPad-based intervention on turn-taking behaviors in preschoolers with autism.*Behavioral Development Bulletin, 20*(2), 253-264. http://dx.doi.org/10.1037/h0101314
61. Knutsen, J., Wolfe, A., Burke, B.L., Hepburn, S., Lindgren, S., & Coury, D. (2016). A systematic review of telemedicine in autism spectrum disorders. *Review of Journal and Developmental Disorders,* 3**,**330–344. https://doi.org/10.1007/s40489-016-0086-9
62. Kobak, K. A., Stone, W. L., Ousley, O. Y., & Swanson, A. (2011). Web-based training in early autism screening: Results from a pilot study.*Telemedicine and e-Health, 17*(8), 640-644. http://dx.doi.org/10.1089/tmj.2011.0029
63. Krysta, K., Krzystanek, M., Cubała, W. J., Wiglusz, M. S., Jakuszkowiak-Wojten, K., Gałuszko-Węgielnik, M., Czarnowska-Cubała, M., Szarmach, J., Włodarczyk, A., & Janas-Kozik, M. (2017). Telepsychiatry and virtual reality in the teatment of patients with intellectual and developmental disabilities.*Psychiatria Danubina, 29*, 656-659. https://search.proquest.com/scholarly-journals/telepsychiatry-virtual-reality-teatment-patients/docview/1992039415/se-2?accountid=14521
64. Kuravackel, G. M., Ruble, L. A., Reese, R. J., Ables, A. P., Rodgers, A. D., & Toland, M. D. (2018). COMPASS for hope: Evaluating the effectiveness of a parent training and support program for children with ASD.*Journal of Autism and Developmental Disorders, 48*(2), 404-416. http://dx.doi.org/10.1007/s10803-017-3333-8
65. Lee, J. F., Schieltz, K. M., Suess, A. N., Wacker, D. P., Romani, P. W., Lindgren, S. D., Kopelman, T. G., & Dalmau, Y. C. (2014). Guidelines for Developing Telehealth Services and Troubleshooting Problems with Telehealth Technology When Coaching Parents to Conduct Functional Analyses and Functional Communication Training in Their Homes. *Behavior analysis in practice*, *8*(2), 190–200. https://doi.org/10.1007/s40617-014-0031-2
66. Lesser, A. D., Luczynski, K. C., & Hood, S. A. (2019). Evaluating motion detection to score sleep disturbance for children: A translational approach to developing a measurement system. *Journal of applied behavior analysis*, *52*(2), 580–599. https://doi.org/10.1002/jaba.531
67. Lindgren, S., Wacker, D., Schieltz, K., Suess, A., Pelzel, K., Kopelman, T., Lee, J., Romani, P., & O'Brien, M. (2020). A randomized controlled trial of functional communication training via telehealth for young children with autism spectrum disorder.*Journal of Autism and Developmental Disorders, 50*(12), 4449-4462. http://dx.doi.org/10.1007/s10803-020-04451-1
68. Little, L. M., Pope, E., Wallisch, A., & Dunn, W. (2018). Occupation-based coaching by means of telehealth for families of young children with autism spectrum disorder.*American Journal of Occupational Therapy, 72*(2)http://dx.doi.org/10.5014/ajot.2018.024786
69. Loman, M., Vogt, E., Miller, L., Landsman, R., Duong, P., Kasten, J., DeFrancisco, D., Koop, J., & Heffelfinger, A. (2021). "How to" operate a pediatric neuropsychology practice during the COVID-19 pandemic: Real tips from one practice's experience. *Child neuropsychology : a journal on normal and abnormal development in childhood and adolescence*, *27*(2), 251–279. https://doi.org/10.1080/09297049.2020.1830962
70. Machalicek, W., O'Reilly, M., Chan, J. M., Rispoli, M., Lang, R., Davis, T., Shogren, K., Sorrells, A., Lancioni, G., Sigafoos, J., Green, V., & Langthorne, P. (2009). Using videoconferencing to support teachers to conduct preference assessments with students with autism and developmental disabilities.*Research in Autism Spectrum Disorders, 3*(1), 32-41. http://dx.doi.org/10.1016/j.rasd.2008.03.004
71. Machalicek, W., Lequia, J., Pinkelman, S., Knowles, C., Raulston, T., Davis, T., & Alresheed, F. (2016). Behavioral telehealth consultation with families of children with autism spectrum disorder.*Behavioral Interventions, 31*(3), 223-250. http://dx.doi.org/10.1002/bin.1450
72. Marcus, S. M., Malas, N. M., Quigley, J. M., Rosenblum, K. L., Muzik, M., LePlatte-Ogini, D. J., & Patel, P. D. (2017). Partnerships with Primary Care for the Treatment of Preschoolers. *Child and adolescent psychiatric clinics of North America*, *26*(3), 597–609. https://doi.org/10.1016/j.chc.2017.03.002
73. Marino, F., Chilà, P., Failla, C., Crimi, I., Minutoli, R., Puglisi, A., Arnao, A. A., Tartarisco, G., Ruta, L., Vagni, D., & Pioggia, G. (2020). Tele-Assisted Behavioral Intervention for Families with Children with Autism Spectrum Disorders: A Randomized Control Trial. *Brain sciences*, *10*(9), 649. https://doi.org/10.3390/brainsci10090649
74. Martino, D., & Hedderly, T. (2019). Tics and stereotypies: A comparative clinical review. *Parkinsonism & related disorders*, *59*, 117–124. https://doi.org/10.1016/j.parkreldis.2019.02.005
75. Max, M.L, & Burke, J.C. (1997). Virtual reality for autism communication and education, with lessons for medical training simulators. *Studies in Health Technology and Informatics*, 39, 46-53.
76. Mazurek, M. O., Parker, R. A., Chan, J., Kuhlthau, K., Sohl, K., & ECHO Autism Collaborative (2020). Effectiveness of the Extension for Community Health Outcomes Model as Applied to Primary Care for Autism: A Partial Stepped-Wedge Randomized Clinical Trial. *JAMA pediatrics*, *174*(5), e196306. https://doi.org/10.1001/jamapediatrics.2019.6306
77. McCarty, C. A., Stoep, A. V., Violette, H., & Myers, K. (2015). Interventions developed for psychiatric and behavioral treatment in the children’s ADHD telemental health treatment study.*Journal of Child and Family Studies, 24*(6), 1735-1743. http://dx.doi.org/10.1007/s10826-014-9977-5
78. McDuffie, A., Machalicek, W., Bullard, L., Nelson, S., Mello, M., Tempero-Feigles, R., Castignetti, N., & Abbeduto, L. (2016). A spoken-language intervention for school-aged boys with fragile X syndrome.*American Journal on Intellectual and Developmental Disabilities, 121*(3), 236-265. http://dx.doi.org/10.1352/1944-7558-121.3.236
79. Miyahara, M., Butson, R., Cutfield, R., & Clarkson, J. E. (2009). A pilot study of family-focused tele-intervention for children with developmental coordination disorder: Development and lessons learned.*Telemedicine and e-Health, 15*(7), 707-712. http://dx.doi.org/10.1089/tmj.2009.0022
80. Narzisi A. (2020). Phase 2 and Later of COVID-19 Lockdown: Is it Possible to Perform Remote Diagnosis and Intervention for Autism Spectrum Disorder? An Online-Mediated Approach. *Journal of clinical medicine*, *9*(6), 1850. https://doi.org/10.3390/jcm9061850
81. Nazneen, N., Matthews, N., Smith, C. J., Rozga, A., Abowd, G. D., Oberleitner, R., . . . Arriaga, R. I. (2015). Use of a novel imaging technology for remote autism diagnosis: A reflection on experience of stakeholders. *Procedia Manufacturing, 3*, 293-300.
82. Neely, L., Rispoli, M., Gerow, S., & Hong, E. R. (2016). Preparing interventionists via telepractice in incidental teaching for children with autism.*Journal of Behavioral Education, 25*(4), 393-416. http://dx.doi.org/10.1007/s10864-016-9250-7
83. Oberleitner, R., Laxminarayan, S., Suri, J., Harrington, J., & Bradstreet, J. (2004). The potential of a store and forward tele-behavioral platform for effective treatment and research of autism. *Conference proceedings : ... Annual International Conference of the IEEE Engineering in Medicine and Biology Society. IEEE Engineering in Medicine and Biology Society. Annual Conference*, *2004*, 3294–3296. https://doi.org/10.1109/IEMBS.2004.1403926
84. Oberleitner, R., Elison-Bowers, P., Reischl, U., & Ball, J. (2007). Optimizing the personal health record with special video capture for the treatment of autism.*Journal of Developmental and Physical Disabilities, 19*(5), 513-518. http://dx.doi.org/10.1007/s10882-007-9067-3
85. Parmanto, B., Pulantara, I. W., Schutte, J. L., Saptono, A., & McCue, M. P. (2013). An integrated telehealth system for remote administration of an adult autism assessment. *Telemedicine journal and e-health : the official journal of the American Telemedicine Association*, *19*(2), 88–94. https://doi.org/10.1089/tmj.2012.0104
86. Parsons, D., Cordier, R., Vaz, S., & Lee, H. C. (2017). Parent-Mediated Intervention Training Delivered Remotely for Children With Autism Spectrum Disorder Living Outside of Urban Areas: Systematic Review. *Journal of medical Internet research*, *19*(8), e198. https://doi.org/10.2196/jmir.6651
87. Pearl, P. L., Sable, C., Evans, S., Knight, J., Cunningham, P., Lotrecchiano, G. R., Gropman, A., Stuart, S., Glass, P., Conway, A., Ramadan, I., Paiva, T., Batshaw, M. L., & Packer, R. J. (2014). International telemedicine consultations for neurodevelopmental disabilities. *Telemedicine journal and e-health: the official journal of the American Telemedicine Association*, *20*(6), 559–562. https://doi.org/10.1089/tmj.2013.0275
88. Peterson, K. M., Ibañez, V. F., Volkert, V. M., Zeleny, J. R., Engler, C. W., & Piazza, C. C. (2021). Using telehealth to provide outpatient follow-up to children with avoidant/restrictive food intake disorder. *Journal of applied behavior analysis*, *54*(1), 6–24. https://doi.org/10.1002/jaba.794
89. Pickard, K. E., Wainer, A. L., Bailey, K. M., & Ingersoll, B. R. (2016). A mixed-method evaluation of the feasibility and acceptability of a telehealth-based parent-mediated intervention for children with autism spectrum disorder.*Autism, 20*(7), 845-855. http://dx.doi.org/10.1177/1362361315614496
90. Pidano, A. E., Slater, C. M., Dale, L. P., Wilbur, K. L., Sandhu, P., & Honigfeld, L. (2016). Availability of telephone-based child psychiatry consultation: Implications from a survey of pediatric providers in two states.*Journal of Child and Family Studies, 25*(8), 2607-2615. http://dx.doi.org/10.1007/s10826-016-0423-8
91. Raman, N., Nagarajan, R., Venkatesh, L., Monica, D. S., Ramkumar, V., & Krumm, M. (2019). School-based language screening among primary school children using telepractice: A feasibility study from India. *International journal of speech-language pathology*, *21*(4), 425–434. https://doi.org/10.1080/17549507.2018.1493142
92. Raspa, M., Fitzgerald, T., Furberg, R. D., Wylie, A., Moultrie, R., DeRamus, M., Wheeler, A. C., & McCormack, L. (2018). Mobile technology use and skills among individuals with fragile X syndrome: Implications for healthcare decision making.*Journal of Intellectual Disability Research, 62*(10), 821-832. http://dx.doi.org/10.1111/jir.12537
93. Reese, R. J., Slone, N. C., Soares, N., & Sprang, R. (2015). Using telepsychology to provide a group parenting program: A preliminary evaluation of effectiveness.*Psychological Services, 12*(3), 274-282. http://dx.doi.org/10.1037/ser0000018
94. Rockhill, C., Violette, H., Stoep, A. V., Grover, S., & Myers, K. (2013). Caregivers' distress: Youth with Attention-Deficit/Hyperactivtty Disorder and comorbid disorders assessed via telemental health.*Journal of Child and Adolescent Psychopharmacology, 23*(6), 379-385. http://dx.doi.org/10.1089/cap.2013.0019
95. Saint-André, S., Neira Zalentein, W., Robin, D., & Lazartigues, A. (2011). La télépsychiatrie au service de l’autisme.*L'Encéphale: Revue De Psychiatrie Clinique Biologique Et Thérapeutique, 37*(1), 18-24. http://dx.doi.org/10.1016/j.encep.2010.03.010
96. Salomone, E., & Maurizio Arduino, G. (2017). Parental attitudes to a telehealth parent coaching intervention for autism spectrum disorder. *Journal of telemedicine and telecare*, *23*(3), 416–420. https://doi.org/10.1177/1357633X16642067
97. Sehlin, H., Hedman Ahlström, B., Andersson, G., & Wentz, E. (2018). Experiences of an internet-based support and coaching model for adolescents and young adults with ADHD and autism spectrum disorder -a qualitative study. *BMC psychiatry*, *18*(1), 15. https://doi.org/10.1186/s12888-018-1599-9
98. Shah, R., Chakrabarti, S., Sharma, A., Grover, S., Sachdeva, D., & Avasthi, A. (2019). Participating from homes and offices: Proof-of-concept study of multi-point videoconferencing to deliver group parent training intervention for attention-deficit/hyperactivity disorder.*Asian Journal of Psychiatry, 41*, 20-22. http://dx.doi.org/10.1016/j.ajp.2019.03.006
99. Shire, S. Y., Worthman, L. B., Shih, W., & Kasari, C. (2020). Comparison of face-to-face and remote support for interventionists learning to deliver JASPER intervention with children who have autism.*Journal of Behavioral Education, 29*(2), 317-338. http://dx.doi.org/10.1007/s10864-020-09376-4
100. Simacek, J., Dimian, A. F., & McComas, J. J. (2017). Communication intervention for young children with severe neurodevelopmental disabilities via telehealth.*Journal of Autism and Developmental Disorders, 47*(3), 744-767. http://dx.doi.org/10.1007/s10803-016-3006-z
101. Sivaraman, M., Virues-Ortega, J., & Roeyers, H. (2021). Telehealth mask wearing training for children with autism during the COVID‐19 pandemic.*Journal of Applied Behavior Analysis, 54*(1), 70-86. http://dx.doi.org/10.1002/jaba.802
102. Sohl, K., Mazurek, M. O., & Brown, R. (2017). ECHO Autism: Using Technology and Mentorship to Bridge Gaps, Increase Access to Care, and Bring Best Practice Autism Care to Primary Care. *Clinical pediatrics*, *56*(6), 509–511. https://doi.org/10.1177/0009922817691825
103. Stainbrook, J. A., Weitlauf, A. S., Juárez, A. P., Taylor, J. L., Hine, J., Broderick, N., Nicholson, A., & Warren, Z. (2019). Measuring the service system impact of a novel telediagnostic service program for young children with autism spectrum disorder.*Autism, 23*(4), 1051-1056. http://dx.doi.org/10.1177/1362361318787797
104. Stiles-Shields, C., Potthoff, L. M., Bounds, D. T., Burns, M., Draxler, J. M., Otwell, C. H., Wolodiger, E. D., Westrick, J., & Karnik, N. S. (2020). Harnessing Phones to Target Pediatric Populations with Socially Complex Needs: Systematic Review. *JMIR pediatrics and parenting*, *3*(2), e19269. https://doi.org/10.2196/19269
105. Storey, K. (2010). Smart houses and smart technology: Overview and implications for independent living and supported living services.*Intellectual and Developmental Disabilities, 48*(6), 464-469. http://dx.doi.org/10.1352/1934-9556-48.6.464
106. Stuckey, R., & Domingues-Montanari, S. (2017). Telemedicine is helping the parents of children with neurodevelopmental disorders living in remote and deprived areas. *Paediatrics and international child health*, *37*(3), 155–157.
107. Sutherland, R., Trembath, D., & Roberts, J. (2018). Telehealth and autism: A systematic search and review of the literature.*International Journal of Speech-Language Pathology, 20*(3), 324-336. http://dx.doi.org/10.1080/17549507.2018.1465123
108. Sutherland, R., Trembath, D., Hodge, M. A., Rose, V., & Roberts, J. (2019). Telehealth and autism: Are telehealth language assessments reliable and feasible for children with autism?*International Journal of Language & Communication Disorders, 54*(2), 281-291. http://dx.doi.org/10.1111/1460-6984.12440
109. Szeftel, R., Federico, C., Hakak, R., Szeftel, Z., & Jacobson, M. (2012). Improved access to mental health evaluation for patients with developmental disabilities using telepsychiatry.*Journal of Telemedicine and Telecare, 18*(6), 317-321. http://dx.doi.org/10.1258/jtt.2012.111113
110. Talbott, M. R., Dufek, S., Zwaigenbaum, L., Bryson, S., Brian, J., Smith, I. M., & Rogers, S. J. (2020). Brief report: Preliminary feasibility of the TEDI: A novel parent-administered telehealth assessment for autism spectrum disorder symptoms in the first year of life.*Journal of Autism and Developmental Disorders, 50*(9), 3432-3439. http://dx.doi.org/10.1007/s10803-019-04314-4
111. Tan-MacNeill, K. M., Smith, I. M., Weiss, S. K., Johnson, S. A., Chorney, J., Constantin, E., Shea, S., Hanlon-Dearman, A., Brown, C. A., Godbout, R., Ipsiroglu, O., Reid, G. J., & Corkum, P. V. (2020). An eHealth insomnia intervention for children with neurodevelopmental disorders: Results of a usability study. *Research in developmental disabilities*, *98*, 103573. https://doi.org/10.1016/j.ridd.2020.103573
112. Tang, J. S. Y., Falkmer, M., Chen, N. T. M., Bölte, S., & Girdler, S. (2021). Development and feasibility of MindChip™: A social emotional telehealth intervention for autistic adults.*Journal of Autism and Developmental Disorders, 51*(4), 1107-1130. http://dx.doi.org/10.1007/s10803-020-04592-3
113. Tariq, Q., Daniels, J., Schwartz, J. N., Washington, P., Kalantarian, H., & Wall, D. P. (2018). Mobiledetection of autism through machine learning on home video: A development and prospective validation study. *PLOS Medicine, 15*(11), e1002705. doi:10.1371/journal.pmed.1002705
114. Tariq, Q., Fleming, S. L., Schwartz, J. N., Dunlap, K., Corbin, C., Washington, P., . . . Wall, D. P. (2019). Detecting developmental delay and autism through machine learning models using home videos of Bangladeshi children: Development and validation study. *Journal of Medical Internet Research, 21*(4), e13822. doi:10.2196/13822
115. Terry M. (2009). Telemedicine and autism: researchers and clinicians are just starting to consider telemedicine applications for the diagnosis and treatment of autism. *Telemedicine journal and e-health : the official journal of the American Telemedicine Association*, *15*(5), 416–419. https://doi.org/10.1089/tmj.2009.9965
116. Tichon, J., & Yellowlees, P. (2003). Internet social support for children and adolescents.*Journal of Telemedicine and Telecare, 9*(4), 238-240. http://dx.doi.org/10.1258/135763303322225599
117. Todorow, C., Connell, J., & Turchi, R. M. (2018). The medical home for children with autism spectrum disorder: an essential element whose time has come. *Current opinion in pediatrics*, *30*(2), 311–317. https://doi.org/10.1097/MOP.0000000000000605
118. Tsami, L., Lerman, D., & Toper‐Korkmaz, O. (2019). Effectiveness and acceptability of parent training via telehealth among families around the world.*Journal of Applied Behavior Analysis, 52*(4), 1113-1129. http://dx.doi.org/10.1002/jaba.645
119. Valentine, A. Z., Hall, S. S., Young, E., Brown, B. J., Groom, M. J., Hollis, C., & Hall, C. L. (2021). Implementation of Telehealth Services to Assess, Monitor, and Treat Neurodevelopmental Disorders: Systematic Review. *Journal of medical Internet research*, *23*(1), e22619. https://doi.org/10.2196/22619
120. van der Gaag, Rutger Jan. (2019). Dépistage précoce et aide à la famille.*Enfance, 2019*(1), 73-81. http://dx.doi.org/10.3917/enf2.191.0073
121. Vismara, L. A., McCormick, C., Young, G. S., Nadhan, A., & Monlux, K. (2013). Preliminary findings of a telehealth approach to parent training in autism. *Journal of autism and developmental disorders*, *43*(12), 2953–2969. https://doi.org/10.1007/s10803-013-1841-8
122. Wacker, D. P., Lee, J. F., Dalmau, Y. C. P., Kopelman, T. G., Lindgren, S. D., Kuhle, J., Pelzel, K. E., & Waldron, D. B. (2013). Conducting functional analyses of problem behavior via telehealth.*Journal of Applied Behavior Analysis, 46*(1), 31-46. http://dx.doi.org/10.1002/jaba.29
123. Washington, P., Leblanc, E., Dunlap, K., Penev, Y., Varma, M., Jung, J. Y., Chrisman, B., Sun, M. W., Stockham, N., Paskov, K. M., Kalantarian, H., Voss, C., Haber, N., & Wall, D. P. (2021). Selection of trustworthy crowd workers for telemedical diagnosis of pediatric autism spectrum disorder. *Pacific Symposium on Biocomputing. Pacific Symposium on Biocomputing*, *26*, 14–25.
124. Whittingham, L. M., & Coons-Harding, K. (2021). Connecting people with people: Diagnosing persons with fetal alcohol spectrum disorder using telehealth.*Journal of Autism and Developmental Disorders, 51*(4), 1067-1080. http://dx.doi.org/10.1007/s10803-020-04607-z
125. Wiederhold, B. K., & Wiederhold, M. D. (2004). The future of cybertherapy: improved options with advanced technologies. *Studies in health technology and informatics*, *99*, 263–270.
126. Xie, Y., Dixon, J. F., Yee, O. M., Zhang, J., Chen, Y. A., DeAngelo, S., Yellowlees, P., Hendren, R., & Schweitzer, J. B. (2013). A study on the effectiveness of videoconferencing on teaching parent training skills to parents of children with ADHD.*Telemedicine and e-Health, 19*(3), 192-199. http://dx.doi.org/10.1089/tmj.2012.0108
